# A site-specific map of the human plasma glycome and its age and gender-associated alterations

**DOI:** 10.1038/s41598-020-73588-x

**Published:** 2020-10-15

**Authors:** Alexander A. Merleev, Dayoung Park, Yixuan Xie, Muchena J. Kailemia, Gege Xu, L. Renee Ruhaak, Kyoungmi Kim, Qiuting Hong, Qiongyu Li, Forum Patel, Yu-Jui Yvonne Wan, Alina I. Marusina, Iannis E. Adamopoulos, Nelvish N. Lal, Anupum Mitra, Stephanie T. Le, Michiko Shimoda, Guillaume Luxardi, Carlito B. Lebrilla, Emanual Maverakis

**Affiliations:** 1 Department of Dermatology, University of California Davis School of Medicine, 3301 C Street Suite 1400, Sacramento, CA 95816 USA; 2 Department of Surgery, Beth Israel Deaconess Medical Center, Harvard Medical School, Boston, MA USA; 3 Department of Chemistry, University of California Davis, One Shields Ave, 2465 Chemistry Annex, Davis, CA 95616 USA; 4 Department of Clinical Chemistry and Laboratory Medicine, Leiden University Medical Center, ZA Leiden, The Netherlands; 5 Division of Biostatistics, Department of Public Health Sciences, University of California Davis, Davis, CA USA; 6 Department of Medical Pathology and Laboratory Medicine, University of California Davis School of Medicine, Sacramento, CA USA; 7 Department of Internal Medicine, Division of Rheumatology, Allergy and Clinical Immunology, University of California Davis School of Medicine, Davis, CA USA; 8 Institute for Pediatric Regenerative Medicine, Shriners Hospitals for Children Northern California, Sacramento, CA USA; 9 Department of Biochemistry and Molecular Medicine, University of California Davis, Davis, CA USA; 10 Foods for Health Institute, University of California Davis, Davis, CA USA

**Keywords:** Glycobiology, Glycomics

## Abstract

Alterations in the human glycome have been associated with cancer and autoimmunity. Thus, constructing a site-specific map of the human glycome for biomarker research and discovery has been a highly sought-after objective. However, due to analytical barriers, comprehensive site-specific glycoprofiling is difficult to perform. To develop a platform to detect easily quantifiable, site-specific, disease-associated glycan alterations for clinical applications, we have adapted the multiple reaction monitoring mass spectrometry method for use in glycan biomarker research. The adaptations allow for highly precise site-specific glycan monitoring with minimum sample prep. Using this technique, we successfully mapped out the relative abundances of the most common 159 glycopeptides in the plasma of 97 healthy volunteers. This plasma glycome map revealed 796 significant (FDR < 0.05) site-specific inter-protein and intra-protein glycan associations, of which the vast majority were previously unknown. Since age and gender are relevant covariants in biomarker research, these variables were also characterized. 13 glycopeptides were found to be associated with gender and 41 to be associated with age. Using just five age-associated glycopeptides, a highly accurate age prediction model was constructed and validated (r^2^ = 0.62 ± 0.12). The human plasma site-specific glycan map described herein has utility in applications ranging from glycan biomarker research and discovery to the development of novel glycan-altering interventions.

## Introduction

Glycans (oligosaccharides) are one of the four fundamental molecules that make up all living systems^1^. Traditionally, proteins are considered the end-product of the information stored in a cell’s genome. However, in order to function appropriately, many proteins require post-translational modifications, and these are commonly glycans. As modifiers, glycans can function as protein “on and off” switches or as “analog regulators” to fine-tune protein function^2^. The process that synthesizes and enzymatically attaches glycans to organic molecules is called glycosylation and it can produce thousands of unique glycan structures by linking together a finite set of sugar monomers^3^. However, unlike DNA, RNA and protein synthesis, there is no template to guide the production of glycans. The process is thus immensely complex and impossible to predict from gene expression profiles alone. In fact, when one considers the massive 3-dimensional structural diversity of glycans combined with their variation in attachment sites, the complexity of the glycome parallels that of the genome^2^.

As part of their glycoscience “Roadmap”^2^, the National Research Council of the U.S. National Academies highlighted the importance of developing a site-specific map of the serum glycome, which would aid in the development of glycans as biomarkers of human diseases. One reason for the excitement around the use of glycans as disease-specific biomarkers is that glycosylation is a process influenced by a variety of factors including: the type of cell and its activation state; environmental factors, such as the presence of available metabolites; the age of the cell, as glycan moieties can be lost over time; and inflammatory mediators, such as cytokines and chemokines. All these factors can be altered in the setting of human diseases, making the glycome an expression of the overall health status of an individual. Furthermore, it has been hypothesized that glycans not only become altered in the setting of human disease but that they actually play a major role in the etiology of all human diseases^2^. It is therefore not surprising that alterations in the glycome have already been linked to a variety of human diseases, especially cancer and autoimmunity^4–16^. Most of these prior studies used labor-intensive methodologies to characterize glycans released from purified proteins and perhaps for this reason, detailed analyses have only been conducted on a relatively small number of patients. Lower resolution techniques, which yield limited structural information or no site-specific information, have been used to characterize larger patient cohorts, but such analyses are not ideally suited for biomarker discovery research. As a result, the sensitivity and specificity of site-specific glycosylations as disease-specific multi-analyte classifiers of autoimmunity is currently unknown.

In comparison to the advances made in the fields of genomics and proteomics, glycoscience remains relatively understudied, which is due to a lack of the analytical tools needed to drive the field forward^2^. In this regard, glycoscience is similar to where the field of genetics was during the initial stages of the human genome project^2^. Mass spectrometry (MS)-based technologies remain very appealing for glycan biomarker research because glycans are ionizable molecules. Also, the potential to accurately profile and quantitate thousands of glycan structures from a relatively small amount of starting material (e.g. 2 µl of serum) makes glycans superior to other molecules traditionally used as biomarkers of human diseases. For example, a site-specific glycoprofiling method could theoretically increase the accuracy of a serum protein biomarker by subdividing it into its different glycoforms.

With the goal of deploying glycan biomarkers clinically, we have developed Multiple Reaction Monitoring (MRM) to site-specifically characterize the human glycome in a rapid and reproducible fashion^17^. Although MRM MS is mainly used in the fields of metabolomics and proteomics^18–21^, its high sensitivity and linear response over a wide dynamic range makes it especially suited for glycan detection^22^. Herein, we employ MRM MS to construct a detailed site-specific structural map of the human plasma glycome of healthy individuals and to characterize the glycans’ inter- and intra-molecular correlations. Glycan alterations associated with age and gender (common covariants in biomarker research and discovery) were also identified and multi-analyte classifiers capable of predicting age were constructed and validated.

## Results

### Site-specific map of the serum glycome in healthy volunteers

**Figure 1:**
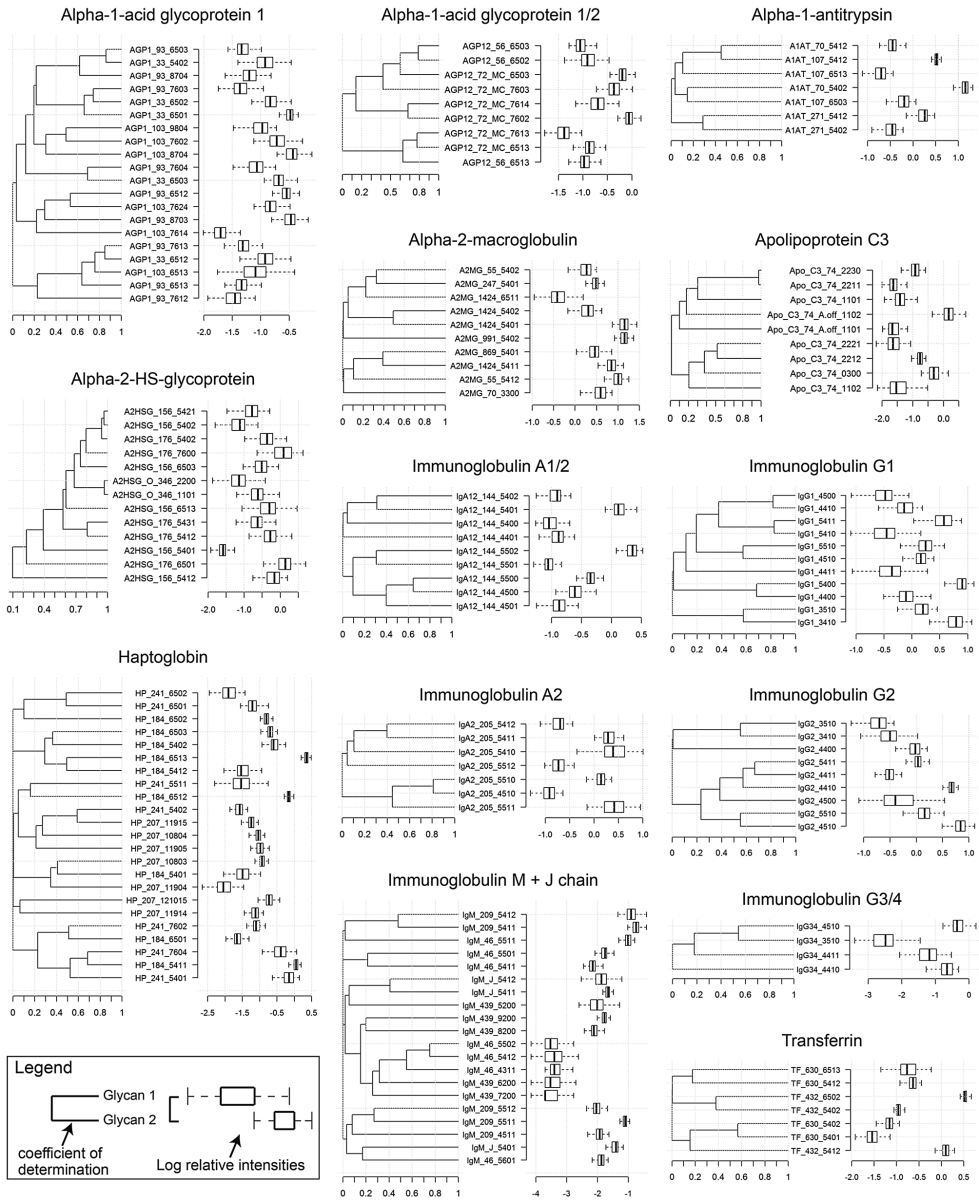
Site-specific map of the human serum glycome. The major glycans occurring at the glycosylations sites of the 17 most common serum glycoproteins are presented. When present, the sites of glycosylation (first of the two numbers) are as indicated in UNIPROT. When there is no position indicated, the glycosylation occurs at the immunoglobulin constant heavy chain domain 2 (CH2)-84.4 glycosylation site (IMGT numbering system). Glycan structures are presented as a four digit code where the first numeral represents the total number of mannose and galactose residues combined, the second represents the total number of N-acetylglucosamine residues, the third numeral corresponds to the number of fucose residues, and the final numeral is the number of sialic acid moieties. On the right side of each diagram is the log of the relative abundances of the glycans presented as box-and-whisker plots. The left and right bars connected to each box indicate the boundaries of the normal distribution and the left and right box edges mark the first and third quartile boundaries within each distribution. The bold line within the box indicates the median value of the distribution. On the left of each diagram are the square of the intra-protein Pearson Product Moment Correlation Coefficients (PPMCCs) for connected glycan pair.

With knowledge of the retention times and collision induced dissociation (CID) behavior of the most abundant serum glycoforms^17,23^ (Table S1), we characterized the relative abundance of 159 glycopeptides (Figs. S1 and S2) within the serum of 97 healthy volunteers with no known history of thyroid disease, cancer, autoimmunity, or other major medical problem. For each glycoprotein, a robustly quantified non-glycosylated peptide (Fig. S1 and S2) was used as an internal reference for calculating each glycoform’s relative abundance. Trypsin-digested protein standards were used to calculate each protein’s absolute abundance. In total, 159 unique glycopeptides were simultaneously monitored (Table S1 and S3) and a site-specific map of the most abundant glycoforms in the human plasma glycome was constructed (Fig. 1).

### Intra- and Inter-protein glycan association

**Figure 2:**
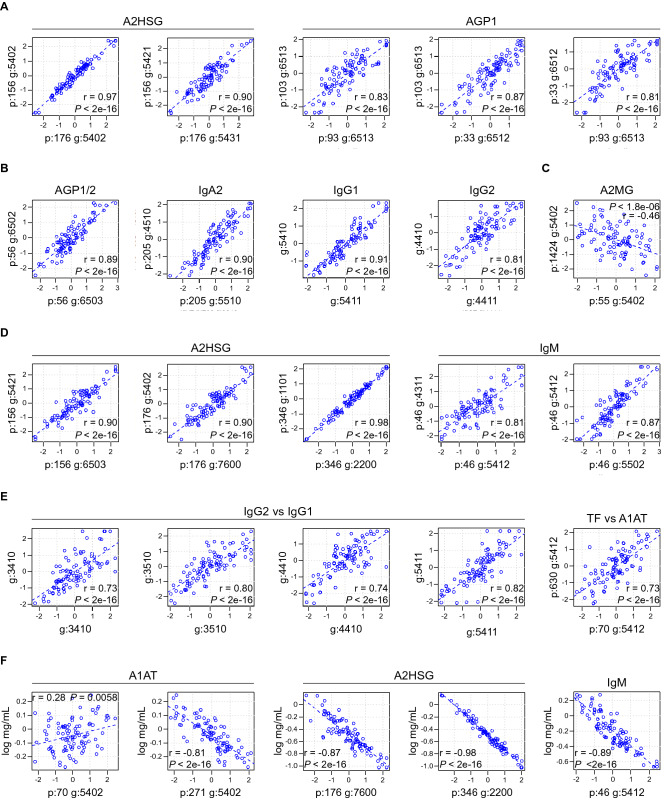
Intra-and inter-protein glycan associations. Log relative abundances for individual glycan pairs were graphed, and correlations were determined using Pearson Product Moment Correlation Coefficients (PPMCCs), which is abbreviated as “r”. (**A**–**D**) are intra-protein correlations. (**E**) represents inter-protein glycan correlations. (**F**) represents protein-glycan correlations. (A comprehensive list of all pairwise correlations between monitored analytes can be found in Data File S1) (n = 97).

Having calculated the relative contribution of each glycopeptide that make up the bulk of the plasma glycome (Fig. 1), we next sought to characterize their inter- and intra-protein relationships (i.e. determine how the presence of one glycan at a particular site correlates with the expression of other glycans at that site and at distant sites within the same or different glycoprotein). For this analysis, we calculated Pearson product-moment correlation coefficients (PPMCCs) for all possible analyte pairs (Figs. 1, 2 and Data File S1). This analysis revealed several distinct types of inter- and intra-protein glycan relationships.

Firstly, it was not uncommon for a glycan at one glycosylation site to positively correlate with the same or highly similar glycans at another distant glycosylation site within the same glycoprotein. In other words, structurally similar glycans often occur at different sites within the same protein. For example, the presence of glycan 5402 (Table S3) at position 176 of Alpha-2-HS-glycoprotein (A2HSG) (Fig. S1) positively correlated (PPMCC 0.974) with the presence of glycan 5402 at site 156 of A2HSG (*P* < 2E−16) (Fig. 2A). Likewise, the presence of glycan 6513 (Table S3) at site 93 of alpha-1-acid glycoprotein (AGP1) (Fig. S1) positively correlated (PPMCC 0.827) with the presence of glycan 6513 at site 103 of AGP1 (*P* < 2E−16) (Fig. 2A). The previously mentioned glycans (6513 at site 93 and 6513 at site 103) also positively correlated (PPMCC’s 0.810 and 0.874, respectively) with a third structurally similar glycan 6512 at site 33 of AGP1 (*P* < 2E−16 for both analyte pairs) (Fig. 2A).

In addition to the same or structurally similar glycans tending to occupy different sites within the same protein, glycans of similar structure also tended to occupy the same glycosylation site. For example, the presence of glycan 5411 (Table S3) strongly correlated (PPMCC 0.908) with glycan 5410 (Table S3) at the same site of IgG1 (*P* < 2E−16) (Fig. 2B). Thus, the glycosylation machinery of a particular cell can drive the appearance of the same or similar glycans across multiple sites within the same protein.

Although the above examples might seem intuitive, the opposite was also possible, i.e. the relative abundance of a glycan at two different sites within the same glycoprotein can be negatively correlated. For example, glycan 5402 at position 55 of A2MG negatively correlated (PPMCC − 0.463) with 5402 at A2MG position 1424 (*P* = 1.84E−06) (Fig. 2C). Thus, in some cases, the cell regulates the presentation of a particular glycan to a specific site, rather than to multiple sites. Finally, there were also examples of structurally distinct glycans residing at the same site positively correlating with one another, an example being glycans 5402 and 7600 (Table S3) which positively correlated (PPMCC 0.900, *P* < 2E−16) with one another at site 176 of alpha 2-HS glycoprotein (A2HSG) (Fig. 2D).

Apart from the intra-protein glycan correlations just described, there were also inter-protein glycan correlations that were of significance [i.e. glycans on different proteins can correlate (positively or negatively) with one another]. This was especially true for the different immunoglobulin subclasses. For example, the abundance of glycan modifiers on IgG1 correlated with their identical counterparts on IgG2 (Figs. 1 and 2E). This is of interest because in theory, IgG1 and IgG2 should be synthesized by different B cell populations, which would indicate that different cells can be influenced to employ similar glycan modifications. Glycan correlations across structurally dissimilar proteins were also sometimes present. One of the most striking of which was the correlation (PPMCC 0.733, *P* < 2E−16) between glycan 5412 at position 70 of Alpha-1 Antitrypsin (A1AT) with glycan 5412 at position 630 of transferrin (TF) (Fig. 2E). Figure S3 is a pictorial representation of the 16,742 correlations (Data File S1) analyzed in this study, of which 796 were significant (FDR < 0.05) (Data File S1). This figure uses a machine learning dimensionality reduction technique to represent the thousands of correlations as a 2D image, where each symbol represents a different site-specific glycosylation. Symbols that are far away from each other correlate poorly, whereas overlapping symbols are highly correlative. From this image, it is clearly apparent that there are both intra- and inter-glycan correlations. Importantly, previous studies of enzymatically cleaved glycans failed to make such distinctions between populations of glycans originating from different proteins.

Finally, in many cases, the relative abundance of a particular glycan at a defined site correlated with the protein’s serum concentration. One interesting example is glycan 5402, which had a small positive correlation (PPMCC 0.28) with A1AT’s serum concentration when present at site A1AT site 70 (*P* = 0.006) but had a strong highly significant negative correlation (PPMCC -0.81) with the serum concentration of A1AT when present at A1AT site 271 (*P* < 2E−16) (Fig. 2F). Other examples were the non-sialylated N-glycan 7600 and O-glycan 2200 (Table S3) occurring at sites 176 and 346 of A2HSG, respectively. Both glycans had a strong negative correlation with A2HSG serum concentration (PPMCC -0.87, *P* < 2E−16, and PPMCC − 0.98, *P* < 2E−16, respectively) (Fig. 2F).

### Analysis of covariates

**Figure 3:**
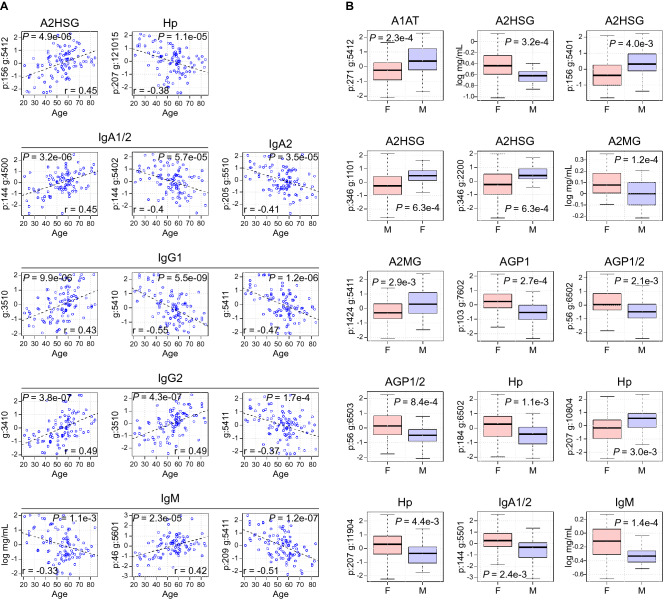
Effect of Age and Gender on glycosylation. (**A**) Log relative glycan abundance versus age. Examples of glycoforms significantly altered by age (a full list can be found in Table S4). Of note, IgG1 and IgG2 share several age-associated glycan modifications. Also, glycan 5411 is negatively correlated with age when present on IgG1, IgG2, and position 209 of IgM. IgM also declines with increasing age (*P* = 0.0011). (**B**) Representative site-specific glycosylations and proteins that are differentially expressed with respect to gender (a full list can be found in Table S5). The upper and lower bars connected to each box indicate the boundaries of the normal distribution and the upper and lower box edges mark the first and third quartile boundaries within each distribution. The bold line within the box indicates the median value of the distribution. Y-axis represents log relative abundance or log protein concentration where indicated.

Previous studies conducted mainly on either released glycans or tryptic peptides of purified IgG have demonstrated that age and gender can alter the glycosylation of serum proteins^24–28^. Thus, we sought to characterize the site-specific glycan alterations that could be contributed to the age and gender effect (Fig. 3, A and B, and Tables S4 and S5). The distribution of age and gender within our healthy control sample set is depicted in Fig. S4, A and B. Plotting relative and absolute abundances against age revealed that increasing age is associated with a modest decline in IgM (PPMCC −0.33) (Fig. 3A). The level of IgM was also affected by gender (FDR = 0.01), with males showing lower plasma levels of IgM than females (0.49 mg/mL [SD 0.2] vs 0.87 mg/mL [SD 0.6], respectively) (Fig. 3B and Table S6). Of the 159 glycopeptides monitored, the intensities of 41 were associated with age (Table S4). Importantly, the specific glycan modifications affected by age were consistent across the different IgG subclasses. For example, for IgG1 and IgG2 subclasses, the non-galactosylated 3510 (Table S3) Fc glycan modification was positively correlated with age (PPMCCs 0.43 and 0.49, respectively) (Fig. 3A). In contrast, the fully galactosylated 5411 at this same site was negatively correlated with age (PPMCCs −0.47 and −0.37, respectively). Interestingly, the similar but non-sialylated IgG1 5410 also negatively correlated with age (PPMCC −0.55, *P* = 5.5e−09) (Fig. 3A). Thus, age-glycan relationships depend on more than just the presence or absence of sialylations, which are traditionally thought to be lost during aging.

Many biological processes are altered by gender and ultimately this leads to differences in disease frequencies and treatment outcomes^29,30^. Thus, characterizing gender-specific alterations in glycosylation is an important step in developing glycans as biomarkers of human disease. Figure 3B reveals that 13 glycopeptides are significantly altered by gender (FDR < 0.05), as were the concentrations of the serum proteins A2HSG, A2MG, and IgM (Fig. 3B and Table S5 and S6). To confirm these results and the age-glycan associations just described above, we next conducted a meta-analysis of 4 healthy control datasets, which allowed us to confirm the observed glycan associations across multiple datasets (Fig. S5 and S6).

### Prediction models for age

**Figure 4:**
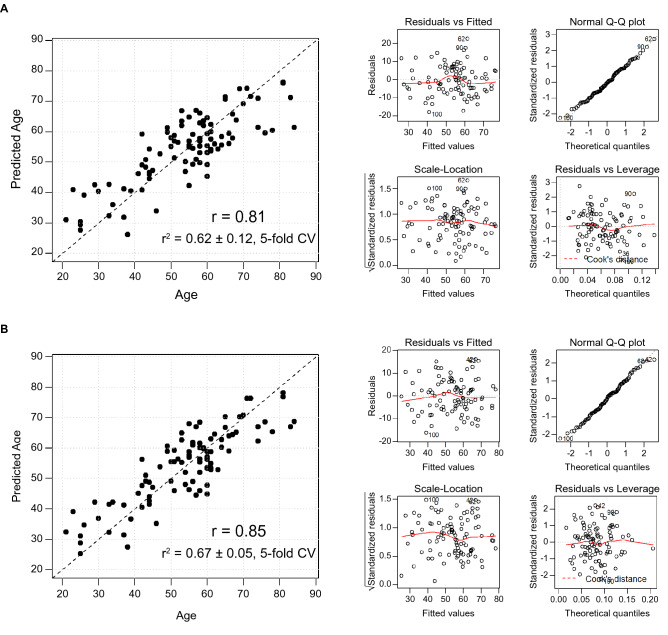
Age Prediction Models. (**A**) The graph represents the performance of a linear regression model for age prediction. The model was constructed from 5 different glycopeptides (IgG1 g:3510, IgG1 g:5410, IgM p:209 g:5411, IgM J chain g:5412, Hp p:241 g:7602). Diagnostic plots (residuals vs fitted, testing for linearity; normal Q-Q, to assess the distribution of the residuals; scale-location, to assess the homoscedastic of the data; and residuals vs leverage, to check for overly influential cases) for the model are presented to its right. (**B**) Linear regression model comprised of six glycopeptides (IgG1 g:3510, IgG1 g:5410, IgG2 g:3410, IgM p:209 g:5411, IgM J chain g:5412, Hp p: 241 g:7602) and 1 serum protein, IgG3. Model diagnostics are represented to the right (model performance parameters for age prediction models can be found in Table S7).

Since there were 41 statistically significant glycopeptides that correlated with age (Table S4), the question arose whether enough information was held within the human glycome to construct an age prediction model. Linear regression models comprised of either glycopeptides only or a mixture of glycopeptides and proteins were thus constructed utilizing a forward stepwise selection method. A resulting “glycan only” model revealed that five sites of glycosylation (IgG1-3510, IgG1-5410, IgM-209-5411, IgM-J-5412, and Haptoglobin (Hp)-241-7602) were sufficient to accurately predict age (PPMCC 0.81) (Fig. 4A and Table S7). Interrogation of the 5-glycopeptide age prediction model revealed low collinearity among its analytes (average variance inflation factor (VIF) = 1.34 ± 0.19) (Table S7) and the diagnostic plots (residuals vs fitted, normal Q-Q, scale-location, and residuals vs leverage) of the model revealed good linearity, normally distributed residuals, homoscedastic data, and a lack of overly influential cases, respectively (Fig. 4A). The multiple fractional polynomial method (MFP) and individual pairwise PPMCCs were also used to evaluate the model constituents for nonlinear relationships and for correlative relationships amongst each other, respectively. These analyses failed to identify nonlinear relationships or meaningful intra-model analyte correlations. Thus, all model diagnostics supported the design of the 5-glycopeptide age prediction model. Finally, the age prediction model was successfully validated using a fivefold cross-validation strategy (r^2^ = 0.62 ± 0.12, fivefold CV) (Table S7).

Because model constituents IgG1-5410 and IgM-J-5412 had been previously monitored, a meta-analysis was also conducted to determine the weighted averages of their respective glycan-age correlations. These meta-analyses yielded averages that were highly significant (*P* < 2E−16 and *P* = 8.4E−06, respectively) with no evidence (*P* = 0.27 and* P* = 0.93, respectively) of any substantial residual heterogeneity (i.e. there was no remaining variability in effect sizes that was unexplained) (Fig. S5).

A second combined age-prediction model, which included serum protein concentrations as additional variables, was also constructed. The resulting model contained six glycopeptides (IgG1-3510, IgG1-5410, IgG2-3410, IgM-209-5411, IgM-J-5412, Hp-241-7602) and 1 serum protein (IgG3). This model was also highly accurate in its ability to predict age (PPMCC 0.85; r^2^ = 0.67 ± 0.05, fivefold CV) (Fig. 4B) and the diagnostic analyses of this combined model revealed similar results as those just described for the “glycan only” model (Fig. 4B and Table S7). Additional prediction models for age (both “glycan only” and “combined”) with differing numbers of variables were also considered and their summary data are presented in Fig. S7 and Table S8. Of note, in each case the performance of the “glycan only” models were similar to their combined model counterparts, which highlights the utility of glycans as biomarkers of complex biological processes, such as aging.

## Discussion

Here we present a detailed site-specific map of the human serum glycome, which reveals many novel features of glycosylation. In some cases, glycosylation varied with protein abundance, such that the probability of a particular site-specific glycosylation occurring became rare as the serum concentration of the protein increased (Fig. 2F). We can speculate on possible mechanisms that could account for this phenomenon. The classic example being asialoglycoprotein receptor recognition of aged non-sialylated proteins. However, our data also revealed examples of sialylated glycans negatively correlating with serum protein concentrations (Fig. 2F). This suggests that multiple mechanisms might target a serum protein for clearance, each serving a different purpose. For example, mechanisms to remove aged glycoproteins are clearly needed and these may be reliant upon non-sialylated proteins being recognized by asialoglycoprotein receptors. However, other scenarios might also impact a glycoprotein’s half-life. Theoretically, when an infection resolves inflammatory mediators should be removed from the circulation. Alternatively, some diseases might negatively impact glycoprotein production. Perhaps there are compensatory mechanisms for low protein production, e.g. increase glycoprotein half-life through altered glycosylation. Of course, the opposite may also be true, disease-related glycan alterations may pathologically signal for the premature clearance of a glycoprotein. Regardless, our results clearly demonstrate that a variety of site-specific glycosylations are associated with glycoprotein serum concentration. From these results, we speculate that site-specific glycosylations can fine-tune the plasma half-life of proteins, i.e. glycoprotein half-life is not merely mediated by age-associated loss of sialylations.

Other interesting phenomena that came to light from our study include the observed correlations of site-specific glycosylations across different proteins. This was especially true for IgG1 and IgG2 glycosylations (Fig. 2F). Evidently, there are global signals that help establish the modifying glycans utilized by different B cell populations (those secreting IgG1 and those secreting IgG2). Likewise, several site-specific glycosylations of unrelated proteins were also found to significantly correlate with one another (Fig. S3). However, the strongest site-specific glycan-glycan correlations were generally within the same protein (Fig. 2). Interestingly, not all glycans occurring at a particular site of glycosylation correlated with one another. Thus, the abundance of some glycans did not influence the abundance of others occurring at the exact same site. Perhaps, different influences dictate the abundance of the non-correlating site-specific glycosylations. Alternatively, the same glycoprotein might be synthesized by different cells or subpopulations of cells, each with their own glycosylation signature. Regardless, it is clear that multiple glycosylation influences are applied to glycosylate the same glycosite.

Importantly, our MRM MS method is substantially different from methods previously employed for analysis of serum IgG glycans^31,32^. Specifically, the prior methods required purification of IgG and enzymatic release of the modifying glycans. Since IgG has multiple sites of glycosylation, analyzing released glycans does not provide site-specific information about the glycans present at the important Ig Fc CH2-84.4 glycosylation site. In contrast, our MRM MS method is site-specific and requires no protein purification. Thus, our glycan mapping results differ significantly from those previously reported^31,32^. Furthermore, some amount of glycan structural information is inevitably lost or altered during the ionization process. Thus, different MS ionization and analysis methods will yield different efficiencies of detection for different glycan structures. Thus, our goal was not to definitively determine the hierarchy of occupancy of a particular glycosylation site. Rather, we set out to develop a highly precise method of site-specific glycan detection (i.e. a method with high reproducibility Figs. S5 and S6). The glycan structures that we monitor can be reproducibly detected in all samples with exceptional test–retest reliability, which will allow for the construction of clinically relevant multi-analyte glycan biomarker models. It also allows us to directly compare how the abundance of a specific glycan at one glycosylation site correlates to the abundance of a glycan at another glycosylation site. This type of analysis is difficult using traditional MS platforms. Highlighting the power of this method, we were able to characterize 16,742 plasma glycan correlations (Fig. S3).

Age and gender are the covariants most commonly accounted for in biomarker research and discovery. As an aid for future glycan biomarker discovery research, we identified the glycan alterations associated with these common covariants. Analysis of a large control group, representing healthy individuals ages 21 to 84 years old, demonstrated that IgM was negatively correlated with age (Fig. 3A), a finding supported by other investigations^33^. In addition, 41 glycopeptides were found to either positively or negatively correlate with age (Table S4). Analysis of the structures of these glycopeptides revealed a positive association between age and a pro-inflammatory glycans (less sialylated glycans and more G0 glycans) but this was not a hard-fast rule, as G0 glycans (biantennary structures that terminate in N-acetylglucosamine residues) did not uniformly increase with age across all glycosylation sites and there were also a few non-G0 glycans that increased with age. An age prediction model revealed that five sites of glycosylation were sufficient to accurately predict the age of 97 individuals (Fig. 4). The exceptional performance of this model to predict age is a testament of how the human plasma glycome is a reflection of human biological processes, in this case, aging. The clinical relevance of our constructed glycan age model is currently unknown, but it is an intriguing possibility that the calculated glycan age is a predictor of one’s natural aging rate, which is obviously different between individuals. Future research into understanding how to alter the human glycome might provide new therapeutic avenues to lower systemic inflammation and possibly even slow aging. The age prediction model(s) we constructed differ dramatically from previous published work on glycan alterations with aging^24–28,34^. Previous models were constructed from released glycans and some were constructed from several glycan “groups”^34^, rather than a small number of site-specific glycosylations.

Our study is unique for a variety of reasons: (1) glycan quantification was site-specific across multiple serum proteins including different Ig classes and subclasses, previous studies typically focus on characterizing released glycans or glycoprofiled only a few serum proteins^4–16,31,32^; (2) the MRM approach employed here eliminates the need for additional protein purification or chemical processing, which allowed for large patient cohorts to be rapidly characterized; (3) the analysis was precise, rapid, and automated for high throughput; (4) it required only 2 µl of serum or plasma and little sample preparation, current techniques require over an mL of blood to quantitate Ig levels; and (5) in addition to total protein quantification (including IgG subtype quantification), the technique provided the relative abundance of each glycopeptide, making it more suitable for biomarker research and discovery. For these reasons, the development of this approach as a clinical diagnostic tool is very appealing, especially when compared to its more labor-intensive alternatives^4–16,31,32^. Since our method is rapidly evolving, with more site-specific glycosylations being incorporated every month, we anticipate that in the near future glycan analysis will become integral to the diagnosis and management of human diseases, especially diseases of the immune system and cancer.

## Material and methods

### Study design

The objective of this study was to identify the relative abundance of site-specific glycosylations of the most abundant plasma proteins and then to use this information to better understand patterns of glycosylation in humans, with the ultimate goal of developing a robust glycan biomarker discovery platform. Healthy individuals were recruited from the University of California (UC) Davis Medical Center. The University of California, Davis Institutional Review Board (Committee B) approved this study. Research was performed in accordance to relevant guidelines and regulations. All participants provided their written informed consent.

### Sample preparation

For each individual enrolled, plasma was separated from whole blood using a Ficoll gradient. From each plasma preparation, a 2 µL aliquot was reduced, alkylated, and then subjected to trypsin digestion at 37 °C^35^. To allow for absolute quantification, 100 µg of each monitored protein (all from Sigma-Aldrich, St. Louis, MO) was digested according to the same protocol and a dilution series was made prior to sample injection.

### UPLC-ESI-QqQ-MS analysis

The neat enzymatically-prepared samples containing both peptides and glycopeptides were then directly analyzed without further hands-on sample cleanup or dilution using an Agilent 1290 infinity liquid chromatography (LC) system coupled to an Agilent 6490 triple quadrupole (QqQ) mass spectrometer (Agilent Technologies, Santa Clara, CA), as previously described^23,35,36^. Briefly, an Agilent Eclipse plus C18 (RRHD 1.8 µm, 2.1 × 100 mm) coupled with an Agilent Eclipse plus C18 pre-column (RRHD 1.8 µm, 2.1 × 5 mm) was used for UPLC separation. 1.0 µL of the digested plasma samples was injected and analyzed using a 25-min binary gradient consisting of solvent A of 3% acetonitrile, 0.1% formic acid, solvent B of 90% acetonitrile, 0.1% formic acid in nano-pure water (v/v) at a flow rate of 0.5 mL/min.

The MRM MS method used for this study requires predetermined knowledge of the peptide or glycopeptide’s LC retention time and its collision induced dissociation (CID) behavior, which we have previously determined for all the non-glycosylated peptides and glycopeptides used in this study (Fig. S8 and Table S1)^17,35,36^. The specific method used herein has been highly validated and the monitored transitions have been described in detail^16,17,36^. Results were integrated using Agilent MassHunter Quantitative Analysis B.5.0 software. Protein concentrations were determined based on calibration curves and glycopeptide relative responses were calculated using the area under the curves of the glycopeptide and a non-glycosylated reference peptide from the same protein.

### Statistical analysis

All statistical analysis were done using R software^37^. For each analyte, skewedness was calculated, and data was log transformed when necessary to remove excessive skewness. Outliers were identified using R package “extreamvalues”^38^, and when present, were winsorized from the analysis, so that the outliers were set equal to the nearest non-outlier value. Analytes could be detected in all samples; thus, there was no need for imputation of missing data. ANCOVA and linear regression assumptions about the normality of residuals were examined by use of the Shapiro–Wilk test. Colinearity of variables in the multivariate models was examined by calculating variance inflation factor (excessive if > 2.5) with R package “car”^39^. Nonlinear relationships between the analytes and the outcome were evaluated with R package “mfp” using a multiple fractional polynomial method^40^. Variable selection in the multiple linear regressions analyses was performed by forward stepwise exhaustive search using “leaps” R package^41^. The algorithm searched the best models of all sizes up to the specified maximum number variables. To identify the best number of variables, each model’s performance was estimated by the leave-one-out cross validation method using “caret”^42^ R package and the number with minimum root-mean-square error (RMSE) was selected. Logistic regression models were fitted using Firth's bias reduction method with the R package “logistf”^43^. This package was also used for automated variable selection based on penalized likelihood ratio tests. Model performance estimated by fivefold cross-validation was calculated using R package "HandTill2001"^44^. Meta-analyses were conducted to assess findings across the multiple datasets using R package “metafor”^45^. A weighted random-effects model was used to estimate a summary effect size. Restricted maximum-likelihood estimator was selected to estimate between-study variance. Weighted estimation with inverse-variance weights was used to fit the model. To present the correlations between all analytes simultaneously, the dimensionality reduction algorithm “t-distributed stochastic neighbor embedding” (t-sne) was used, implemented in the R package “Rtsne”^46^.

## Supplementary information

Supplementary Information 1.

Supplementary Information 2.

Supplementary Dataset.

## Data Availability

All data are provided in the manuscript and the Supplementary Materials.
