# Supplementary material for: A site-specific map of the human plasma glycome and its age and gender-associated alterations
Source: Sci Rep. 2020 Oct 15;10:17505. doi: 10.1038/s41598-020-73588-x (PMC7567094; doi:10.1038/s41598-020-73588-x)
Supplement: Supplementary file 1 — Supplementary Information 1. [file 41598_2020_73588_MOESM1_ESM.pdf]

A site-specific map of the human plasma glycome and its age and gender-associated alterations

Alexander A. Merleev[a], Dayoung Park[b,c], Yixuan Xie[c], Muchena J. Kailemia[c], Gege Xu[c], L. Renee Ruhaak[c,d], Kyoungmi Kim[e], Qiuting Hong[c], Qiongyu Li[c], Forum Patel[a], Yu-Jui Yvonne Wan[f], Alina I. Marusina[a], Iannis E. Adamopoulos[g,h], Nelvish N. Lal[a], Anupum Mitra[e], Stephanie T. Le[a], Michiko Shimoda[a], Guillaume Luxardi[a], Carlito B. Lebrilla[c,i,j,*], Emanual Maverakis[a,*]

[a]Department of Dermatology, University of California Davis School of Medicine, Sacramento, CA, USA.
[b]Department of Surgery, Beth Israel Deaconess Medical Center, Harvard Medical School, Boston, MA, USA.
[c]Department of Chemistry, University of California Davis, Davis, CA, USA.
[d]Department of Clinical Chemistry and Laboratory Medicine, Leiden University Medical Center, ZA Leiden, The N-etherlands.
[e]Department of Medical Pathology and Laboratory Medicine, University of California Davis School of Medicine, Sacramento, CA, USA.
[f]Division of Biostatistics, Department of Public Health Sciences, University of California Davis, Davis, CA, USA.
[g]Department of Internal Medicine, Division of Rheumatology, Allergy and Clinical Immunology, University of California Davis School of Medicine, Davis, CA, USA.
[h]Institute for Pediatric Regenerative Medicine, Shriners Hospitals for Children Northern California, Sacramento, CA, USA.
[i]Department of Biochemistry and Molecular Medicine, University of California Davis, Davis, CA, USA.
[j]Foods for Health Institute, University of California Davis, Davis, CA, USA.

*Corresponding Authors:
Carlito B. Lebrilla
One Shields Ave
Chemistry Department
2465 Chemistry Annex
Davis, CA 95616
Tel: (530) 752-6364
Email: cblebrilla@ucdavis.edu

Emanual Maverakis
Department of Dermatology
University of California, Davis
3301 C Street Suite 1400
Sacramento, CA 95816
Tel: (916) 551-2631
Email: emaverakis@ucdavis.edu

Keywords: N-glycopeptides, Multiple Reaction Monitoring, Aging, Site-specific glycans

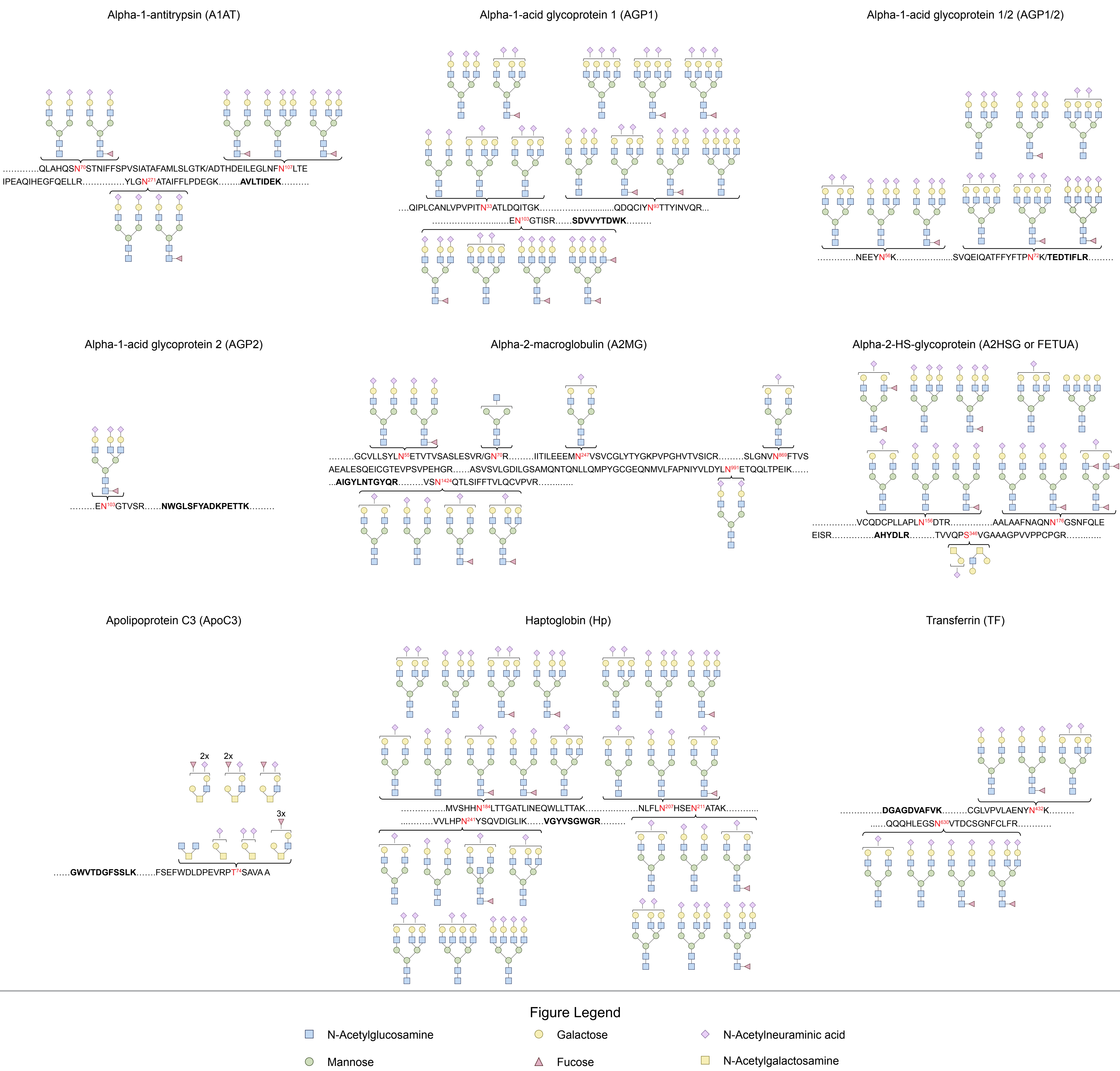

Alpha-1-antitrypsin (A1AT)
............QLAHQSN70STNIFFSPVSIATAFAMLSLGTK/ADTHDEILEGLNFN107LTE
IPEAQIHEGFQELLR...............YLGN271ATAIFFLPDEGK........AVLTIDEK..........
Alpha-1-acid glycoprotein 1 (AGP1)
....QIPLCANLVPVPITN33ATLDQITGK.........................QDQCIYN93TTYINVQR...
..........................EN103GTISR......SDVVYTDWK.........
Alpha-1-acid glycoprotein 1/2 (AGP1/2)
.............NEEYN56K....................SVQEIQATFFYFTPN72K/TEDTIFLR.........
Alpha-1-acid glycoprotein 2 (AGP2)
.........EN103GTVSR......NWGLSFYADKPETTK.........
Alpha-2-macroglobulin (A2MG)
.........GCVLLSYLN55ETVTVSASLESVR/GN70R.........IITILEEEMN247VSVCGLYTYGKPVPGHVTVSICR.........SLGNVN869FTVS
AEALESQEICGTEVPSVPEHGR......ASVSVLGDILGSAMQNTQNLLQMPYGCGEQNMVLFAPNIYVLDYLN991ETQQLTPEIK......
...AIGYLNTGYQR.........VSN1424QTLSIFFTVLQCVPVR..........
Alpha-2-HS-glycoprotein (A2HSG or FETUA)
................VCQDCPLLAPLN156DTR..............AALAAFNAQNN176GSNFQLE
EISR...............AHYDLR.........TVVQPS346VGAAAGPVVPPCPGR............
Apolipoprotein C3 (ApoC3)
2x
2x
3x
......GWVTDGFSSLK.......FSEFWDLDPEVRPT74SAVA A
Haptoglobin (Hp)
................MVSHHN184LTTGATLINEQWLLTTAK.....................NLFLN207HSEN211ATAK...........
............VVLHPN241YSQVDIGLIK......VGYVSGWGR.........
Transferrin (TF)
...............DGAGDVAFVK.........CGLVPVLAENYN432K.........
......QQQHLEGSN630VTDCSGNFCLFR............
Figure Legend
N-Acetylglucosamine
Galactose
N-Acetylneuraminic acid
Mannose
Fucose
N-Acetylgalactosamine

Fig. S1. Site specific map of the most common glycan modifications of the most common serum glycoproteins (excluding immunoglobulins). Putative structures and locations of the site-specific glycans that were monitored in this study. The structures represent the most common glycans occurring at each glycosylation site. Some glycosylation sites can be expressed without a modifying glycan, in which case the non-glycosylated version was also monitored. For each protein a non-glycosylated reference peptide, bolded sequence, present across all glycoforms was used to calculate the relative abundance of each glycoform (i.e. area under the curve of the glycoform divided by the area under the curve of the non-glycosylated reference peptide).

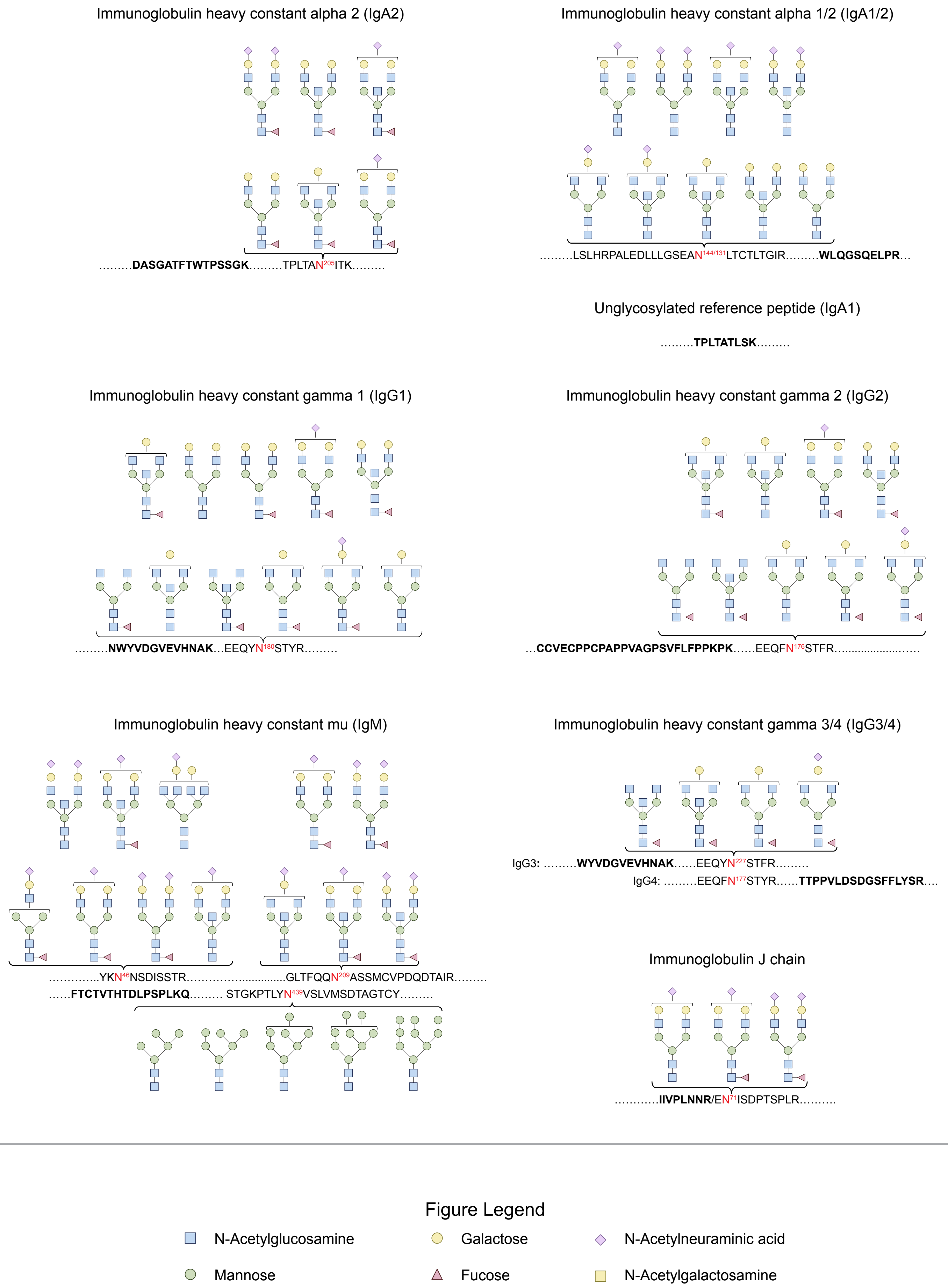

Immunoglobulin heavy constant alpha 2 (IgA2)
.........DASGATFTWTPSSGK.........TPLTAN205ITK.........
Immunoglobulin heavy constant alpha 1/2 (IgA1/2)
.........LSLHRPALEDLLLGSEAN144/131LTCTLTGIR.........WLQGSQELPR...
Unglycosylated reference peptide (IgA1)
.........TPLTATLSK.........
Immunoglobulin heavy constant gamma 1 (IgG1)
.........NWYVDGVEVHNAK...EEQYN180STYR.........
Immunoglobulin heavy constant gamma 2 (IgG2)
...CCVECPPCPAPPVAGPSVFLFPPKPK......EEQFN176STFR.............................
Immunoglobulin heavy constant mu (IgM)
..............YKN46NSDISSTR.............................GLTFQQN209ASSMCVPDQDTAIR.........
......FTCTVTHTDLPSPLKQ......... STGKPTLYN439VSLVMSDTAGTCY.........
Immunoglobulin heavy constant gamma 3/4 (IgG3/4)
IgG3: .........WYVDGVEVHNAK......EEQYN227STFR.........
IgG4: ....EEQFN177STYR......TTPPVLDSDGSFFLYSR....
Immunoglobulin J chain
...........IIVPLNNR/EN71ISDPTSPLR..........
Figure Legend
N-Acetylglucosamine
Galactose
N-Acetylneuraminic acid
Mannose
Fucose
N-Acetylgalactosamine

Fig. S2. Site specific glycan map for the Immunoglobulins (Igs). The CH2 84.4 Ig glycosylation site is conserved across all IgG subclasses (IgG1-4). Glycans at this site and other sites across the different Ig classes (IgA, IgG, IgM, and J chain) were monitored. To provide the relative abundance of each IgG subclass (IgG1-4) the abundance of subclass-specific non-glycosylated peptides were calculated relative to a single non-glycosylated peptide common to all IgG subclasses (IgG1-4). In addition, glycosylated peptides within each subclass were determined relative to a non-glycosylated peptide common to all glycoforms. For IgG3 and IgG4 the glycosylated peptides amino acid sequence was identical, unable to distinguish between the two similar Ig subclasses. Thus, glycosylated peptides from this region are referred to as IgG3/4.

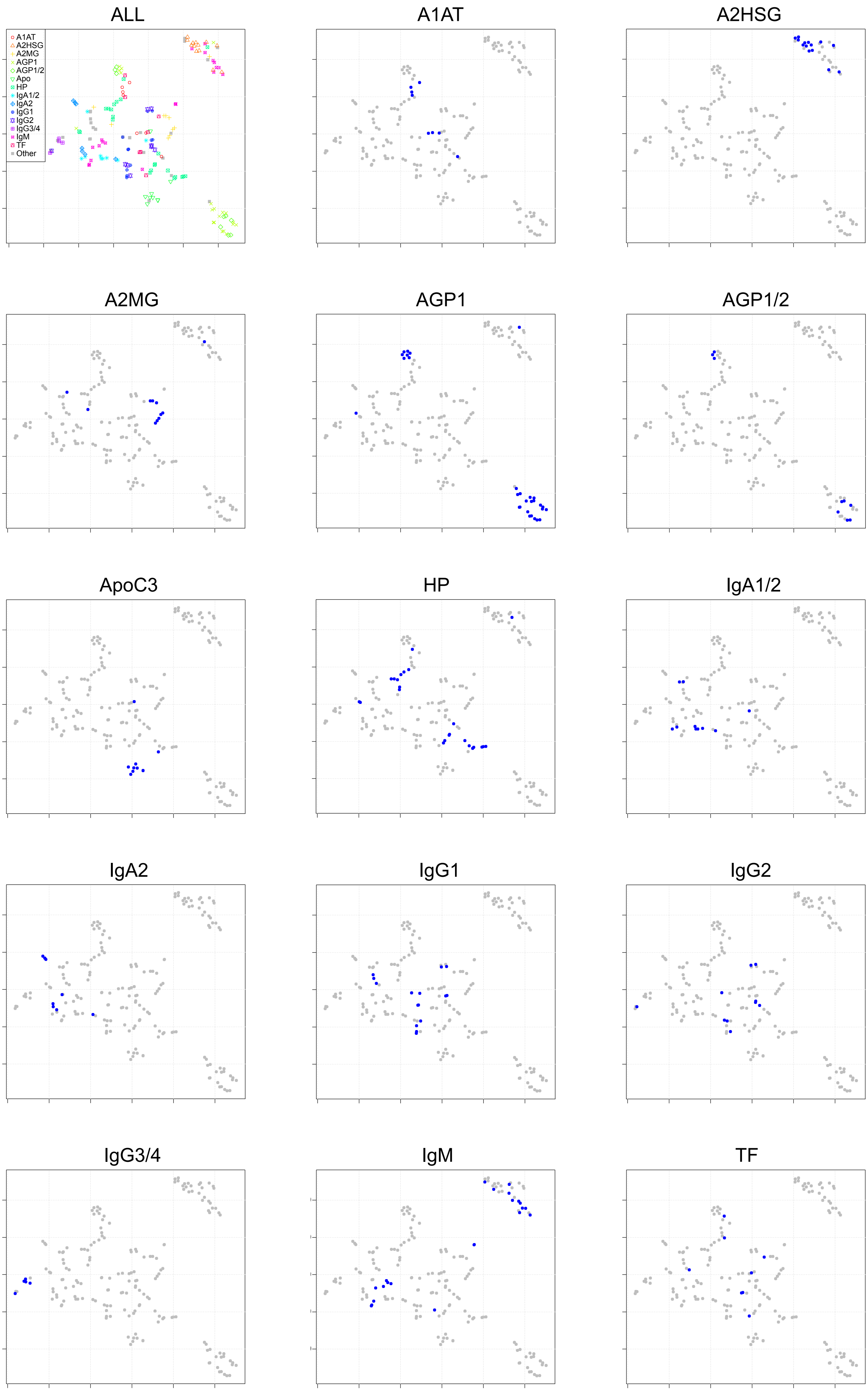
ALL
A1AT
A2HSG
A2MG
AGP1
AGP1/2
Apo
HP
IgA1/2
IgA2
IgG1
IgG2
IgG3/4
IgM
TF
Other
A1AT
A2HSG
A2MG
AGP1
AGP1/2
ApoC3
HP
IgA1/2
IgA2
IgG1
IgG2
IgG3/4
IgM
TF

Fig. S3. Site-specific inter-protein and intra-protein glycan associations. To visualize the 16,742 correlations presented in Data File S1, a machine learning dimensionality reduction strategy, t-Distributed Stochastic Neighbor Embedding, was used. Individual glycosylation sites are represented as distinct symbols. Each copy of the symbol represents a unique glycan occurring at that site. The distance between any two symbols represents the strength of the glycan pair's Pearson Product Moment Correlation Coefficient such that strongly correlating glycans are located close to each other. From this diagram it is apparent that there are both intra-protein and intra-protein glycan correlations. In addition, correlations are grouped into clusters indicating that not all glycosylation sites within a protein correlate with one another.

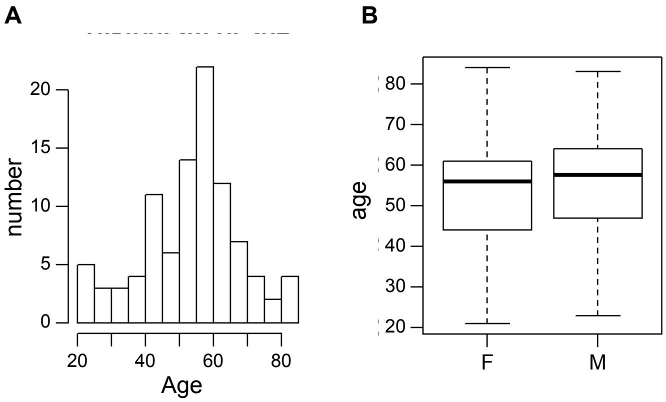

Fig. S4. Age and gender distribution of participants. (**A**) Histogram of age distribution for healthy controls. (**B**) Box plot of age distribution by gender within the healthy control group.

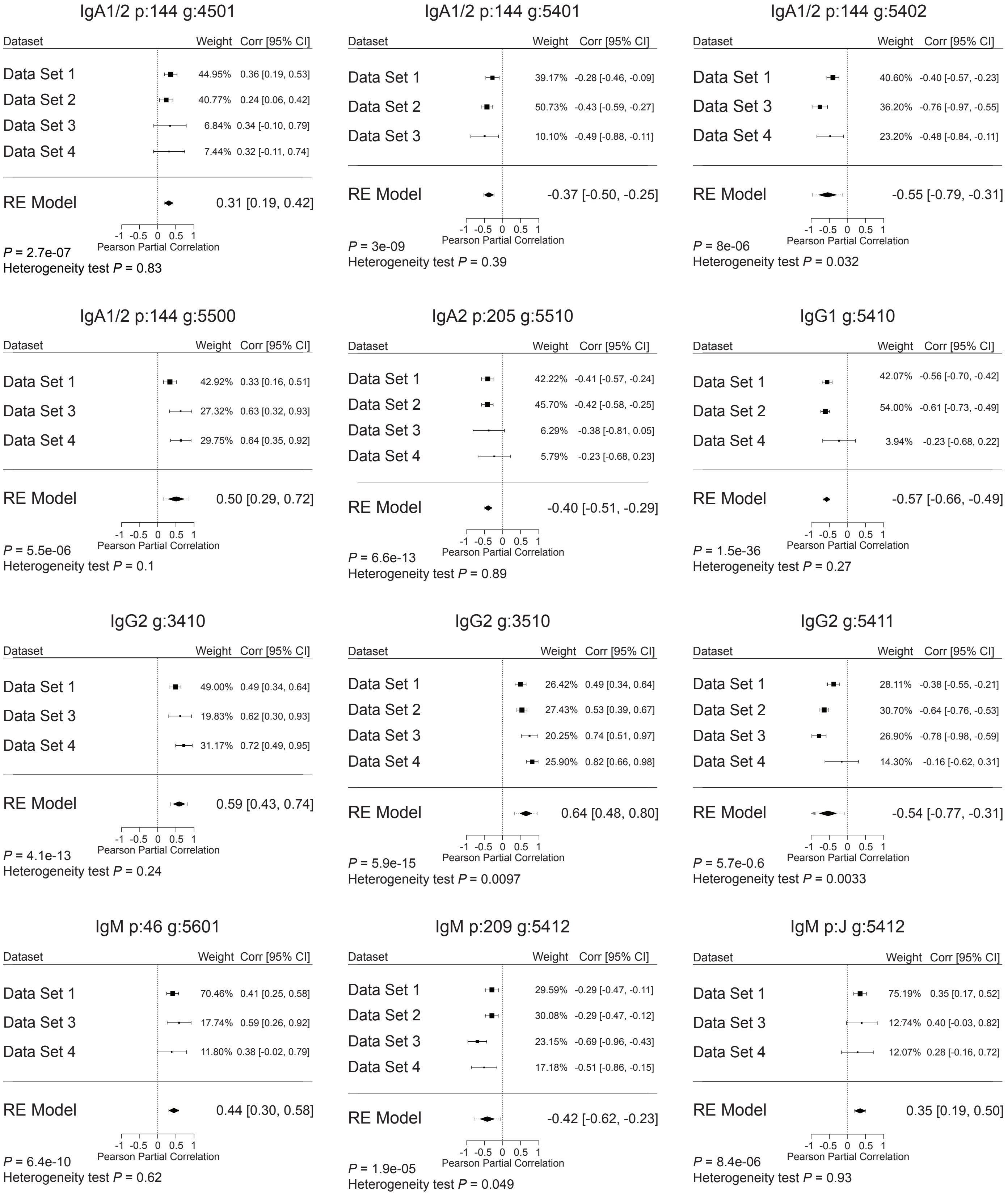

Fig. S5. Metanalysis of glycan associations with age. Forest plots were generated to estimate the Pearson Product Moment Correlation Coefficients (which is abbreviated as "r") between the relative abundances of the indicated glycans and age. In these plots the confidence interval for each dataset is represented by the horizontal lines and the area of each square is proportional to the study's weight in the metanalysis. The final random effects models (RE model) represent the weighted average of the glycan correlations across the different independent data sets and 95% confidence intervals are provided for the given glycan's correlation with age. In each presented case, the confidence interval did not cross zero, although in 4 out of the 12 cases (IgA1/2 p:144 g:5402, IgG2 g:3510, IgG2 g:5411, and IgM p:209 g:5412) the residual heterogeneity was significant, meaning that the variation in glycan age correlations between datasets was high.

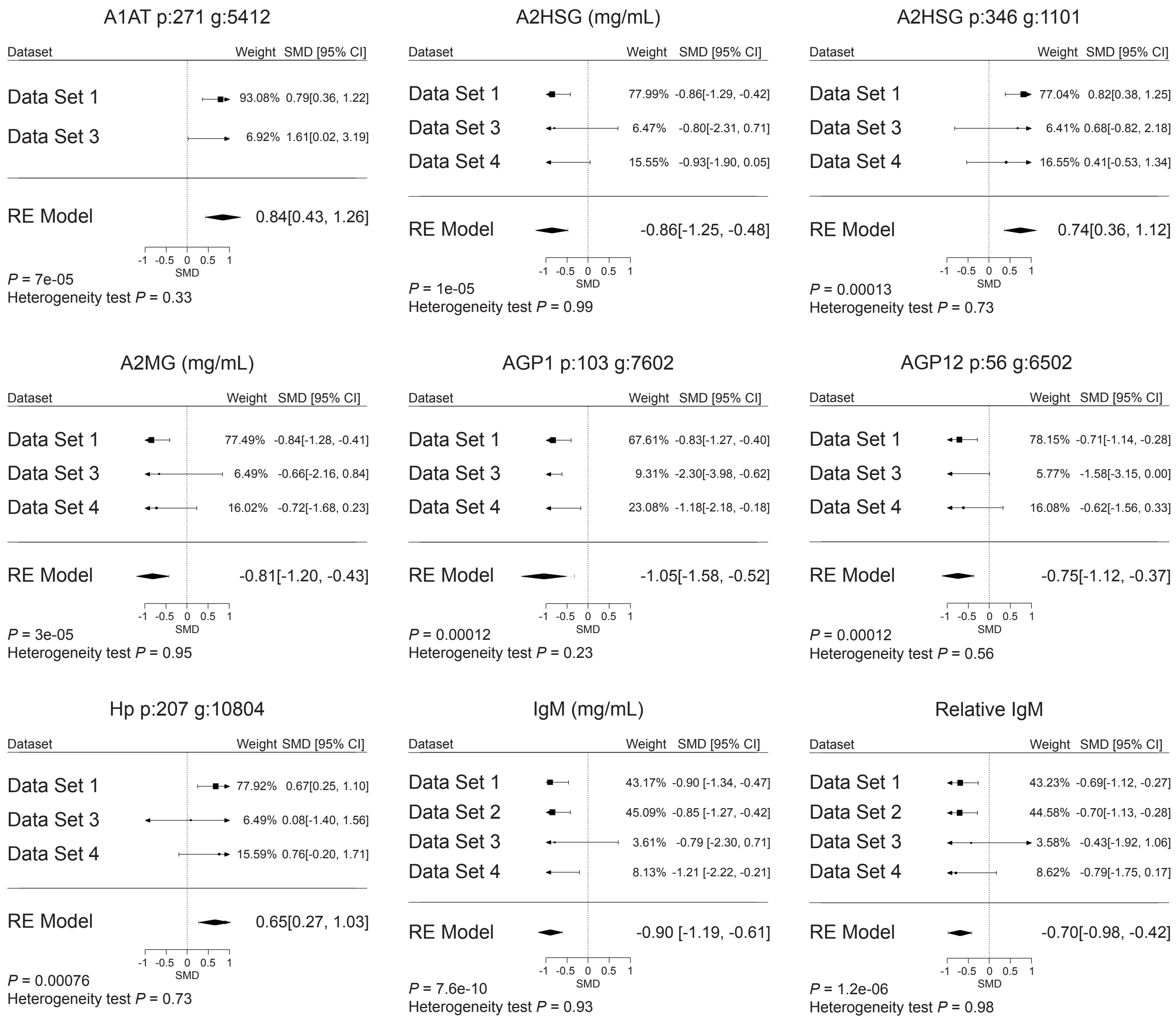

Fig. S6. Meta-analysis of glycan associations with gender. Forest plots were generated to estimate the relative abundance of the indicated glycans or proteins across gender. In each case a final Random effects model (RE model) was constructed to represent the weighted average and 95% confidence interval for a given glycan's abundance. In each presented case the confidence interval did not cross zero and in all cases the residual heterogeneity was not statistically significant. In these plots the confidence interval for each dataset is represented by the horizontal lines and the area of each square is proportional to the study's weight in the meta-analysis.

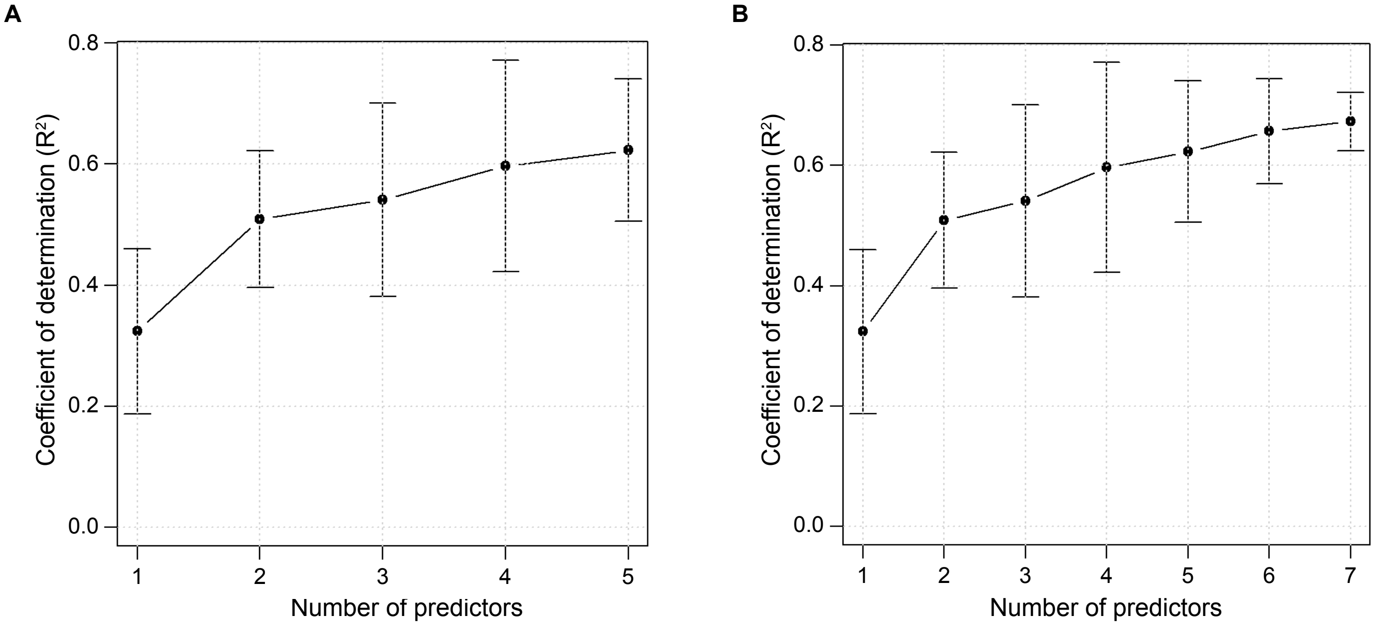

Fig. S7. Performance of age models with differing number of predictors (n). (A) Linear regression model performance improved with incorporation of additional glycans until 5 glycans were incorporated. **(B)** The performance of the linear regression model comprised of both glycoforms and serum protein concentrations improved until 7 analytes were incorporated. n = 7 was chosen as the final model. A comprehensive list of all analytes included in the age prediction models can be found in Table S8.

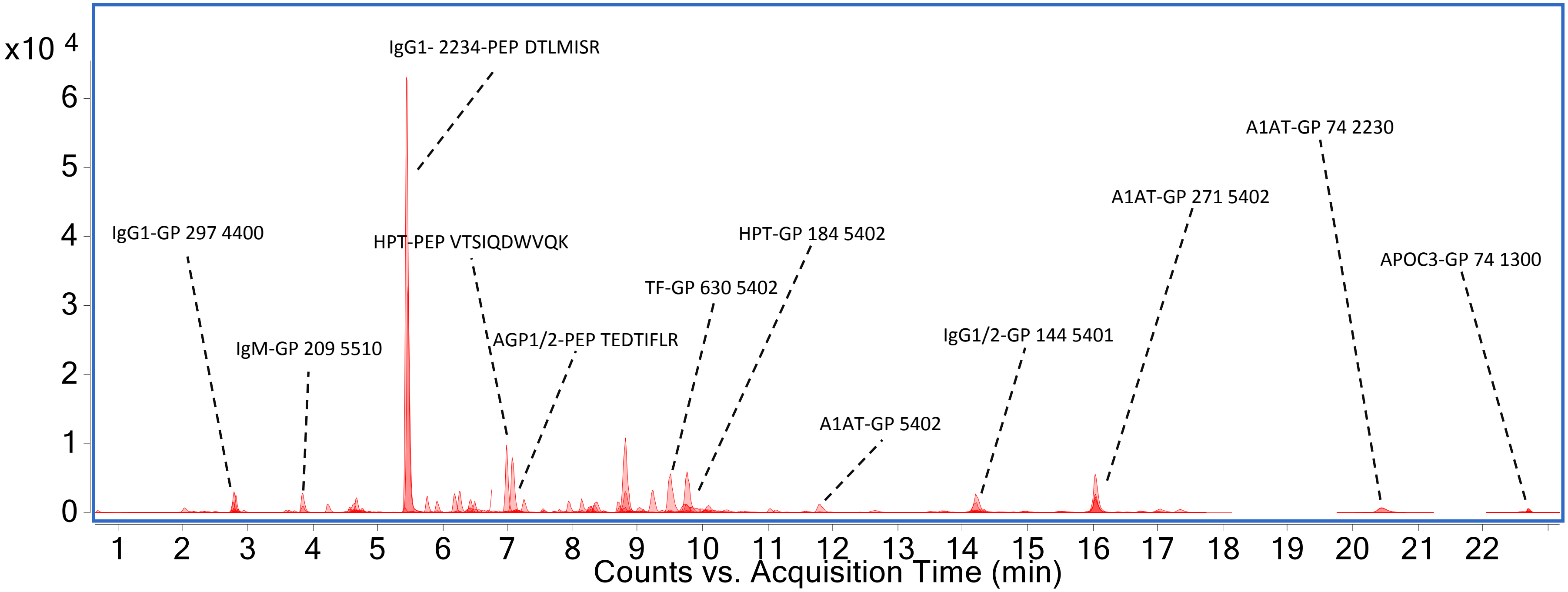

Fig. S8. Dynamic multiple reaction monitoring mass spectrometry (MRM MS). Spectrum generated by QQQ mass spectrometry are shown. The MRM MS technique is dependent on predetermined knowledge of each glycopeptide's retention time and its collision-induced dissociation (CID) pattern (Table S1). The development of the annotated libraries containing this information have been well described 17,35,36. Knowledge of the CID pattern and analyte retention time allows for single transition monitoring of over 1000 specific glycopeptides. Representative compounds are shown.
