# Supplementary material for: A site-specific map of the human plasma glycome and its age and gender-associated alterations
Source: Sci Rep. 2020 Oct 15;10:17505. doi: 10.1038/s41598-020-73588-x (PMC7567094; doi:10.1038/s41598-020-73588-x)
Supplement: Supplementary file 2 — Supplementary Information 2. [file 41598_2020_73588_MOESM2_ESM.docx]

**A site-specific map of the human plasma glycome and its age and gender-associated alterations**

Alexander A. Merleev^a^, Dayoung Park^b,c^, Yixuan Xie^c^, Muchena J. Kailemia^c^, Gege Xu^c^, L. Renee Ruhaak^c,d^, Kyoungmi Kim^e^, Qiuting Hong^c^, Qiongyu Li^c^, Forum Patel^a^, Yu-Jui Yvonne Wan^f^, Alina I. Marusina^a^, Iannis E. Adamopoulos^g,h^, Nelvish N. Lal^a^, Anupum Mitra^e^, Stephanie T. Le^a^, Michiko Shimoda^a^, Guillaume Luxardi^a^, Carlito B. Lebrilla^c,i,j,*^, Emanual Maverakis^a,*^

^a^Department of Dermatology, University of California Davis School of Medicine, Sacramento, CA, USA.

^b^Department of Surgery, Beth Israel Deaconess Medical Center, Harvard Medical School, Boston, MA, USA.

^c^Department of Chemistry, University of California Davis, Davis, CA, USA.

^d^Department of Clinical Chemistry and Laboratory Medicine, Leiden University Medical Center, ZA Leiden, The N­­­etherlands.

^e^Department of Medical Pathology and Laboratory Medicine, University of California Davis School of Medicine, Sacramento, CA, USA.

^f^Division of Biostatistics, Department of Public Health Sciences, University of California Davis, Davis, CA, USA.

^g^Department of Internal Medicine, Division of Rheumatology, Allergy and Clinical Immunology, University of California Davis School of Medicine, Davis, CA, USA.

^h^Institute for Pediatric Regenerative Medicine, Shriners Hospitals for Children Northern California, Sacramento, CA, USA.

^i^Department of Biochemistry and Molecular Medicine, University of California Davis, Davis, CA, USA.

^j^Foods for Health Institute, University of California Davis, Davis, CA, USA.

*Corresponding Authors:

Carlito B. Lebrilla

One Shields Ave

Chemistry Department

2465 Chemistry Annex

Davis, CA 95616

Tel: (530) 752-6364

Email: cblebrilla@ucdavis.edu

Emanual Maverakis

Department of Dermatology

University of California, Davis

3301 C Street Suite 1400

Sacramento, CA 95816

Tel: (916) 551-2631

Email: [emaverakis@ucdavis.edu](mailto:emaverakis@ucdavis.edu)

**Keywords:** N-glycopeptides, Multiple Reaction Monitoring, Aging, Site-specific glycans

| **Supplementary Table 1. Multiple Reaction Monitoring Mass Spectrometry (MRM MS)- Monitored Transitions** | | | | | | |
| --- | --- | --- | --- | --- | --- | --- |
| ***Cpd Name*** | **Ion Monitored** | **Frag (V)** | **CE (V)** | **Cell Acc (V)** | **Ret Time (min)** | **Polarity** |
| *A1AT_107_5402* | 1180.57->366.1 | 380 | 30 | 5 | 12.2 | Positive |
| *A1AT_107_5411* | 1151.56->366.1 | 380 | 30 | 5 | 16 | Positive |
| *A1AT_107_5412* | 1209.78->366.1 | 380 | 30 | 5 | 16.5 | Positive |
| *A1AT_107_6503* | 1311.82->366.1 | 380 | 30 | 5 | 17 | Positive |
| *A1AT_107_6513* | 1341.03->366.1 | 380 | 30 | 5 | 17 | Positive |
| *A1AT_271_5402* | 991.2->366.1 | 380 | 30 | 5 | 11.9 | Positive |
| *A1AT_271_5412* | 1027.71->366.1 | 380 | 30 | 5 | 11.9 | Positive |
| *A1AT_271_MC_5402* | 1149.93->366.1 | 380 | 30 | 5 | 16 | Positive |
| *A1AT_271_MC_5412* | 1179.14->366.1 | 380 | 30 | 5 | 13.8 | Positive |
| *A1AT_70_5402* | 1078.49->366.1 | 380 | 30 | 5 | 20.5 | Positive |
| *A1AT_70_5412* | 1107.7->366.1 | 380 | 30 | 5 | 20.5 | Positive |
| *A2HSG_ Peptide* | 360.1->519.3 | 380 | 4 | 5 | 0.75 | Positive |
| *A2HSG_ Peptide* | 360.1->289.1 | 380 | 4 | 5 | 0.75 | Positive |
| *A2HSG_156_5401* | 1229.18->366.1 | 380 | 20 | 5 | 8.2 | Positive |
| *A2HSG_156_5402* | 994.9->366.1 | 380 | 21 | 5 | 8.2 | Positive |
| *A2HSG_156_5412* | 1374.89->366.1 | 380 | 22 | 5 | 7.2 | Positive |
| *A2HSG_156_5421* | 1326.55->366.1 | 380 | 21 | 5 | 8.2 | Positive |
| *A2HSG_156_6502* | 1086.19->366.1 | 380 | 17 | 5 | 8.2 | Positive |
| *A2HSG_156_6503* | 1158.97->366.1 | 380 | 18 | 5 | 8.2 | Positive |
| *A2HSG_156_6510* | 1234.85->366.1 | 380 | 20 | 5 | 9 | Positive |
| *A2HSG_156_6513* | 1195.48->366.1 | 380 | 19 | 5 | 7.1 | Positive |
| *A2HSG_176_5401* | 1070.4->366.1 | 380 | 17 | 5 | 9 | Positive |
| *A2HSG_176_5402* | 1142.99->366.1 | 380 | 18 | 5 | 9 | Positive |
| *A2HSG_176_5412* | 1179.7->366.1 | 380 | 19 | 5 | 9 | Positive |
| *A2HSG_176_5431* | 1180.26->366.1 | 380 | 19 | 5 | 9 | Positive |
| *A2HSG_176_6501* | 1161.7->366.1 | 380 | 19 | 5 | 10.5 | Positive |
| *A2HSG_176_6502* | 1234.27->366.1 | 380 | 20 | 5 | 9.9 | Positive |
| *A2HSG_176_6503* | 1307.05->366.1 | 380 | 21 | 5 | 8.2 | Positive |
| *A2HSG_176_6512* | 1271.03->366.1 | 380 | 20 | 5 | 9 | Positive |
| *A2HSG_176_6513* | 1343.81->366.1 | 380 | 22 | 5 | 9 | Positive |
| *A2HSG_176_7600* | 1180.5->366.1 | 380 | 19 | 5 | 9 | Positive |
| *A2HSG_O_319_1101* | 913.0865->274.09 | 380 | 25 | 5 | 22.8 | Positive |
| *A2HSG_O_319_1111* | 961.779->274.09 | 380 | 25 | 5 | 22.8 | Positive |
| *A2HSG_O_346_1101* | 891.44->274.09 | 380 | 25 | 5 | 22.8 | Positive |
| *A2HSG_O_346_2110* | 897.11->366.1 | 380 | 25 | 5 | 22.8 | Positive |
| *A2HSG_O_346_2200* | 916.12->366.1 | 380 | 25 | 5 | 22.8 | Positive |
| *A2HSG_Peptide* | 387.69->566.3 | 380 | 5 | 5 | 4 | Positive |
| *A2HSG_Peptide* | 387.6->288.2 | 380 | 5 | 5 | 4 | Positive |
| *A2MG_1424_5401* | 1020.3->366.1 | 380 | 30 | 5 | 17 | Positive |
| *A2MG_1424_5402* | 1093.08->366.1 | 380 | 30 | 5 | 17.4 | Positive |
| *A2MG_1424_5411* | 1056.82->366.1 | 380 | 30 | 5 | 17 | Positive |
| *A2MG_1424_5412* | 1129.59->366.1 | 380 | 30 | 5 | 17 | Positive |
| *A2MG_1424_6501* | 1111.59->366.1 | 380 | 30 | 5 | 17 | Positive |
| *A2MG_1424_6511* | 1148.1->366.1 | 380 | 30 | 5 | 14.2 | Positive |
| *A2MG_247_5200* | 1239.21->1314.16 | 380 | 28 | 5 | 12.9 | Positive |
| *A2MG_247_5401* | 1131.02->366.1 | 380 | 30 | 5 | 12.9 | Positive |
| *A2MG_247_5402* | 1189.24->366.1 | 380 | 30 | 5 | 12.2 | Positive |
| *A2MG_55_5401* | 1078.86->366.1 | 380 | 30 | 5 | 15 | Positive |
| *A2MG_55_5402* | 1151.63->366.1 | 380 | 30 | 5 | 16 | Positive |
| *A2MG_55_5411* | 1115.37->366.1 | 380 | 30 | 5 | 15 | Positive |
| *A2MG_55_5412* | 1188.15->366.1 | 380 | 30 | 5 | 15.5 | Positive |
| *A2MG_70_3300* | 721.39->204.1 | 380 | 30 | 5 | 2.2 | Positive |
| *A2MG_70_5401* | 1130.53->366.1 | 380 | 30 | 5 | 2.2 | Positive |
| *A2MG_70_5402* | 1276.07->366.1 | 380 | 30 | 5 | 2.2 | Positive |
| *A2MG_70_5411* | 1203.55->366.1 | 380 | 30 | 5 | 2.2 | Positive |
| *A2MG_70_5412* | 1349.1->366.1 | 380 | 30 | 5 | 2.2 | Positive |
| *A2MG_70_6511* | 1386.12->366.1 | 380 | 30 | 5 | 2.2 | Positive |
| *A2MG_869_5200* | 1158.79->1206.94 | 380 | 27 | 5 | 10 | Positive |
| *A2MG_869_5401* | 1066.68->366.1 | 380 | 30 | 5 | 10 | Positive |
| *A2MG_869_5402* | 1124.9->366.1 | 380 | 30 | 5 | 10 | Positive |
| *A2MG_869_6200* | 1199.3->1206.94 | 380 | 27 | 5 | 10 | Positive |
| *A2MG_869_7200* | 1239.82->1206.94 | 380 | 26 | 5 | 10 | Positive |
| *A2MG_991_5402* | 1206.28->366.1 | 380 | 30 | 5 | 22.8 | Positive |
| *AGP1_103_6503* | 1213.28->366.1 | 380 | 30 | 5 | 2.2 | Positive |
| *AGP1_103_6513* | 1261.97->366.1 | 380 | 30 | 5 | 2.2 | Positive |
| *AGP1_103_7602* | 1237.96->366.1 | 380 | 30 | 5 | 2.2 | Positive |
| *AGP1_103_7603* | 1334.99->366.1 | 380 | 30 | 5 | 2.2 | Positive |
| *AGP1_103_7604* | 1074.27->366.1 | 380 | 30 | 5 | 2.2 | Positive |
| *AGP1_103_7612* | 1286.64->366.1 | 380 | 30 | 5 | 2.2 | Positive |
| *AGP1_103_7613* | 1383.68->366.1 | 380 | 30 | 5 | 2.2 | Positive |
| *AGP1_103_7614* | 1110.78->366.1 | 380 | 30 | 5 | 2.2 | Positive |
| *AGP1_103_7624* | 1147.3->366.1 | 380 | 30 | 5 | 2.2 | Positive |
| *AGP1_103_8703* | 1092.78->366.1 | 380 | 30 | 5 | 2.2 | Positive |
| *AGP1_103_8704* | 1165.55->366.1 | 380 | 30 | 5 | 2.2 | Positive |
| *AGP1_103_9804* | 1256.84->366.1 | 380 | 30 | 5 | 2.2 | Positive |
| *AGP1_33_5402* | 1196.46->366.1 | 380 | 30 | 5 | 7.2 | Positive |
| *AGP1_33_6501* | 1214.97->366.1 | 380 | 30 | 5 | 7 | Positive |
| *AGP1_33_6502* | 1287.74->366.1 | 380 | 30 | 5 | 7 | Positive |
| *AGP1_33_6503* | 1088.61->366.1 | 380 | 30 | 5 | 7.2 | Positive |
| *AGP1_33_6512* | 1324.26->366.1 | 380 | 30 | 5 | 7 | Positive |
| *AGP1_33_6513* | 1117.83->366.1 | 380 | 30 | 5 | 7.2 | Positive |
| *AGP1_33_7603* | 1161.64->366.1 | 380 | 30 | 5 | 6.1 | Positive |
| *AGP1_93_6502* | 1122.51->366.1 | 380 | 30 | 5 | 7.2 | Positive |
| *AGP1_93_6503* | 1195.28->366.1 | 380 | 30 | 5 | 7.1 | Positive |
| *AGP1_93_6512* | 1159.02->366.1 | 380 | 30 | 5 | 8.2 | Positive |
| *AGP1_93_6513* | 1231.8->366.1 | 380 | 30 | 5 | 7.1 | Positive |
| *AGP1_93_7602* | 1213.79->366.1 | 380 | 30 | 5 | 7.1 | Positive |
| *AGP1_93_7603* | 1286.56->366.1 | 380 | 30 | 5 | 7.1 | Positive |
| *AGP1_93_7604* | 1087.67->366.1 | 380 | 30 | 5 | 7.2 | Positive |
| *AGP1_93_7612* | 1250.3->366.1 | 380 | 30 | 5 | 7 | Positive |
| *AGP1_93_7613* | 1323.08->366.1 | 380 | 30 | 5 | 7.1 | Positive |
| *AGP1_93_7614* | 1116.88->366.1 | 380 | 30 | 5 | 7.5 | Positive |
| *AGP1_93_8703* | 1102.48->366.1 | 380 | 30 | 5 | 7.5 | Positive |
| *AGP1_93_8704* | 967.42->366.1 | 380 | 30 | 5 | 7.1 | Positive |
| *AGP1_93_8713* | 1131.69->366.1 | 380 | 30 | 5 | 8 | Positive |
| *AGP12_56_5402* | 1001.2->366.1 | 380 | 30 | 5 | 1.9 | Positive |
| *AGP12_56_6502* | 1122.91->366.1 | 380 | 30 | 5 | 2.1 | Positive |
| *AGP12_56_6503* | 1219.94->366.1 | 380 | 30 | 5 | 2.1 | Positive |
| *AGP12_56_6513* | 1268.63->366.1 | 380 | 30 | 5 | 2.1 | Positive |
| *AGP2_103_6503* | 1208.6->366.1 | 380 | 30 | 5 | 2.1 | Positive |
| *AGP2_103_6513* | 1257.29->366.1 | 380 | 30 | 5 | 2.1 | Positive |
| *AGP2_103_7603* | 1330.32->366.1 | 380 | 30 | 5 | 2.1 | Positive |
| *AGP2_103_7613* | 1379->366.1 | 380 | 30 | 5 | 4 | Positive |
| *Apo _C3_74_0300* | 916.09->204.1 | 380 | 14 | 5 | 10 | Positive |
| *Apo_C3_74_0310* | 975.44->204.1 | 380 | 15 | 5 | 11.5 | Positive |
| *Apo_C3_74_1101* | 931.76->274.09 | 380 | 14 | 5 | 11.9 | Positive |
| *Apo_C3_74_1102* | 1028.79->274.09 | 380 | 16 | 5 | 12 | Positive |
| *Apo_C3_74_1111* | 980.44->274.1 | 380 | 15 | 5 | 10.5 | Positive |
| *Apo_C3_74_1202* | 1096.48->274.1 | 380 | 17 | 5 | 11.5 | Positive |
| *Apo_C3_74_1210* | 951.1->366.1 | 380 | 15 | 5 | 22.8 | Positive |
| *Apo_C3_74_1300* | 970.1->366.1 | 380 | 15 | 5 | 22.8 | Positive |
| *Apo_C3_74_1311* | 837.13->274.1 | 380 | 13 | 5 | 11.5 | Positive |
| *Apo_C3_74_2200* | 956.43->366.1 | 380 | 15 | 5 | 22.8 | Positive |
| *Apo_C3_74_2211* | 1102.15->274.1 | 380 | 17 | 5 | 17 | Positive |
| *Apo_C3_74_2212* | 899.63->274.1 | 380 | 14 | 5 | 13 | Positive |
| *Apo_C3_74_2220* | 1053.8->366.1 | 380 | 17 | 5 | 7.8 | Positive |
| *Apo_C3_74_2221* | 1150.84->274.1 | 380 | 18 | 5 | 16 | Positive |
| *Apo_C3_74_2230* | 1078.8->366.1 | 380 | 17 | 5 | 20.5 | Positive |
| *Apo_Peptide 1* | 598.8->854.4 | 380 | 8 | 5 | 8.8 | Positive |
| *Apo_Peptide 1* | 598.8->244.1 | 380 | 8 | 5 | 8.8 | Positive |
| *Apo_Peptide 2* | 449.71->434.3 | 380 | 6 | 5 | 6 | Positive |
| *Apo_Peptide 2* | 449.7->251.1 | 380 | 6 | 5 | 6 | Positive |
| *Apo_Peptide 3* | 1069->1097.5 | 380 | 17 | 5 | 11 | Positive |
| *Apo_Peptide 3* | 1069->772.4 | 380 | 17 | 5 | 11 | Positive |
| *C3_85_5200* | 1158.34->1230.34 | 380 | 33 | 5 | 8 | Positive |
| *C3_85_6200* | 909.52->1230.34 | 380 | 22 | 5 | 8 | Positive |
| *C3_85_7200* | 950.03->1230.34 | 380 | 22 | 5 | 8 | Positive |
| *H2HSG_O_319_1101* | 913.1->274.1 | 380 | 25 | 5 | 10.8 | Positive |
| *H2HSG_O_319_1102* | 757.8417->274.1 | 380 | 25 | 5 | 9 | Positive |
| *H2HSG_O_319_1111* | 961.779->274.1 | 380 | 25 | 5 | 9 | Positive |
| *H2HSG_O_319_1201* | 735.8445->274.1 | 380 | 25 | 5 | 9 | Positive |
| *H2HSG_O_346_1102* | 988.4697->274.1 | 380 | 25 | 5 | 16.8 | Positive |
| *HP_184_5401* | 1149.4->366.1 | 380 | 30 | 5 | 9.2 | Positive |
| *HP_184_5402* | 1222.2->366.1 | 380 | 30 | 5 | 9.9 | Positive |
| *HP_184_5411* | 1186->366.1 | 380 | 30 | 5 | 8.5 | Positive |
| *HP_184_5412* | 1258.7->366.1 | 380 | 30 | 5 | 9.8 | Positive |
| *HP_184_6501* | 992.8->366.1 | 380 | 30 | 5 | 9.2 | Positive |
| *HP_184_6502* | 1051->366.1 | 380 | 30 | 5 | 9.8 | Positive |
| *HP_184_6503* | 1109.2->366.1 | 380 | 30 | 5 | 10.1 | Positive |
| *HP_184_6512* | 1080.2->366.1 | 380 | 30 | 5 | 8.1 | Positive |
| *HP_184_6513* | 1138.4->366.1 | 380 | 30 | 5 | 10.1 | Positive |
| *HP_184_7602* | 1124->366.1 | 380 | 30 | 5 | 11.3 | Positive |
| *HP_207_5401* | 1116.4->366.1 | 380 | 30 | 5 | 4.6 | Positive |
| *HP_207_5411* | 1174.6->366.1 | 380 | 30 | 5 | 4.7 | Positive |
| *HP_207_5402* | 1247.7->366.1 | 380 | 30 | 5 | 4.7 | Positive |
| *HP_207_6502* | 1305.9->366.1 | 380 | 30 | 5 | 4.7 | Positive |
| *HP_207_6503* | 1276.9->366.1 | 380 | 30 | 5 | 4.7 | Positive |
| *HP_207_6513* | 1335.1->366.1 | 380 | 30 | 5 | 8.8 | Positive |
| *HP_241_5401* | 1237.3->366.1 | 380 | 30 | 5 | 6.5 | Positive |
| *HP_241_5402* | 1001->366.1 | 380 | 30 | 5 | 8.8 | Positive |
| *HP_241_5412* | 1383->366.1 | 380 | 30 | 5 | 8.8 | Positive |
| *HP_241_5511* | 1015.5->366.1 | 380 | 30 | 5 | 8.8 | Positive |
| *HP_241_6501* | 1019.5->366.1 | 380 | 15 | 5 | 8.3 | Positive |
| *HP_241_6502* | 1092.3->366.1 | 380 | 30 | 5 | 8.7 | Positive |
| *HP_241_6503* | 1165->366.1 | 380 | 30 | 5 | 9 | Positive |
| *HP_241_6512* | 1128.8->366.1 | 380 | 30 | 5 | 8.2 | Positive |
| *HP_241_6513* | 1201.5->366.1 | 380 | 30 | 5 | 7.1 | Positive |
| *HP_241_7602* | 1183.5->366.1 | 380 | 30 | 5 | 8.4 | Positive |
| *HP_241_7603* | 1256.3->366.1 | 380 | 30 | 5 | 11 | Positive |
| *HP_241_7604* | 1063.5->366.1 | 380 | 30 | 5 | 8.1 | Positive |
| *HP_241_7613* | 1292.8->366.1 | 380 | 30 | 5 | 7.1 | Positive |
| *IgA12_144_3500* | 1117.1->366.1 | 380 | 25 | 5 | 13.6 | Positive |
| *IgA12_144_4401* | 943.9->366.1 | 380 | 20 | 5 | 14.3 | Positive |
| *IgA12_144_4500* | 1157.6->366.1 | 380 | 25 | 5 | 14.2 | Positive |
| *IgA12_144_4501* | 1230.4->366.1 | 380 | 30 | 5 | 14.3 | Positive |
| *IgA12_144_5400* | 1147.3->366.1 | 380 | 25 | 5 | 14.2 | Positive |
| *IgA12_144_5401* | 976.3->366.1 | 380 | 25 | 5 | 14.2 | Positive |
| *IgA12_144_5402* | 1292.9->366.1 | 380 | 30 | 5 | 15 | Positive |
| *IgA12_144_5500* | 1198.1->366.1 | 380 | 25 | 5 | 13.7 | Positive |
| *IgA12_144_5501* | 1016.9->366.1 | 380 | 25 | 5 | 14.2 | Positive |
| *IgA12_144_5502* | 1075.1->366.1 | 380 | 25 | 5 | 15.5 | Positive |
| *IgA2_205_4510* | 923.5->366.1 | 380 | 25 | 5 | 4.6 | Positive |
| *IgA2_205_5410* | 909.8->366.1 | 380 | 18 | 5 | 4.8 | Positive |
| *IgA2_205_5411* | 1006.8->366.1 | 380 | 25 | 5 | 4.8 | Positive |
| *IgA2_205_5412* | 1103.8->366.1 | 380 | 25 | 5 | 5 | Positive |
| *IgA2_205_5510* | 977.5->366.1 | 380 | 19 | 5 | 4.6 | Positive |
| *IgA2_205_5511* | 1074.5->366.1 | 380 | 25 | 5 | 4.8 | Positive |
| *IgA2_205_5512* | 878.9->366.1 | 380 | 17 | 5 | 5 | Positive |
| *IgG1 Peptide* | 624.99->1042.55 | 380 | 30 | 5 | 6.6 | Positive |
| *IgG1 Peptide* | 624.99->521.77 | 380 | 30 | 5 | 6.6 | Positive |

**Supplementary Table 2. List of all analytes monitored**

| 1. A1AT (mg/mL) 2. A1AT p:107 g:5412 3. A1AT p:107 g:6503 4. A1AT p:107 g:6513 5. A1AT p:271 g:5402 6. A1AT p:271 g:5412 7. A1AT p:70 g:5402 8. A1AT p:70 g:5412 9. A2HSG (mg/mL) 10. A2HSG p:156 g:5401 11. A2HSG p:156 g:5402 12. A2HSG p:156 g:5412 13. A2HSG p:156 g:5421 14. A2HSG p:156 g:6503 15. A2HSG p:156 g:6513 16. A2HSG p:176 g:5402 17. A2HSG p:176 g:5412 18. A2HSG p:176 g:5431 19. A2HSG p:176 g:6501 20. A2HSG p:176 g:7600 21. A2HSG p:346 g:1101 22. A2HSG p:346 g:2200 23. A2MG (mg/mL) 24. A2MG p:1424 g:5401 25. A2MG p:1424 g:5402 26. A2MG p:1424 g:5411 27. A2MG p:1424 g:6511 28. A2MG p:247 g:5401 29. A2MG p:55 g:5402 30. A2MG p:55 g:5412 31. A2MG p:70 g:3300 32. A2MG p:869 g:5401 33. A2MG p:991 g:5402 34. AGP (mg/mL) 35. AGP1 p:103 8704 36. AGP1 p:103 g: 6513 37. AGP1 p:103 g:7602 38. AGP1 p:103 g:7614 39. AGP1 p:103 g:7624 40. AGP1 p:103 g:9804 41. AGP1 p:33 g:5402 42. AGP1 p:33 g:6501 43. AGP1 p:33 g:6502 44. AGP1 p:33 g:6503 45. AGP1 p:33 g:6512 46. AGP1 p:93 g:6503 | 1. AGP1 p:93 g:6512 2. AGP1 p:93 g:6513 3. AGP1 p:93 g:7603 4. AGP1 p:93 g:7604 5. AGP1 p:93 g:7612 6. AGP1 p:93 g:7613 7. AGP1 p:93 g:8703 8. AGP1 p:93 g:8704 9. AGP1/2 p:56 g:6502 10. AGP1/2 p:56 g:6503 11. AGP1/2 p:56 g:6513 12. AGP1/2 p:72MC g:6503 13. AGP1/2 p:72MC g:6513 14. AGP1/2 p:72MC g:7602 15. AGP1/2 p:72MC g:7603 16. AGP1/2 p:72MC g:7613 17. AGP1/2 p:72MC g:7614 18. AGP2 p:103 g:6513 19. ApoC3 (mg/mL) 20. ApoC3 p:74 g:0300 21. ApoC3 p:74 g:1101 22. ApoC3 p:74 g:1102 23. ApoC3 p:74 g:2211 24. ApoC3 p:74 g:2212 25. ApoC3 p:74 g:2221 26. ApoC3 p:74 g:2230 27. ApoC3 p:74A.off g:1101 28. ApoC3 p:74A.off g:1102 29. Hp (mg/mL) 30. Hp p:184 g:5401 31. Hp p:184 g:5402 32. Hp p:184 g:5411 33. Hp p:184 g:5412 34. Hp p:184 g:6501 35. Hp p:184 g:6502 36. Hp p:184 g:6503 37. Hp p:184 g:6512 38. Hp p:184 g:6513 39. Hp p:207 g:10803 40. Hp p:207 g:10804 41. Hp p:207 g:11904 42. Hp p:207 g:11905 43. Hp p:207 g:11914 44. Hp p:207 g:11915 45. Hp p:207 g:121015 46. Hp p:241 g:5401 | 1. Hp p:241 g:5402 2. Hp p:241 g:5511 3. Hp p:241 g:6501 4. Hp p:241 g:6502 5. Hp p:241 g:7602 6. Hp p:241 g:7604 7. IgA (mg/mL) 8. IgA1 Norm Resp 9. IgA1/2 p:144 4501 10. IgA1/2 p:144 g:4401 11. IgA1/2 p:144 g:4500 12. IgA1/2 p:144 g:5400 13. IgA1/2 p:144 g:5401 14. IgA1/2 p:144 g:5402 15. IgA1/2 p:144 g:5500 16. IgA1/2 p:144 g:5501 17. IgA1/2 p:144 g:5502 18. IgA2 Norm Resp 19. IgA2 p:205 g:4510 20. IgA2 p:205 g:5410 21. IgA2 p:205 g:5411 22. IgA2 p:205 g:5412 23. IgA2 p:205 g:5510 24. IgA2 p:205 g:5511 25. IgG (mg/mL) 26. IgG1 g:3410 27. IgG1 g:3510 28. IgG1 g:4400 29. IgG1 g:4410 30. IgG1 g:4411 31. IgG1 g:4500 32. IgG1 g:4510 33. IgG1 g:5400 34. IgG1 g:5410 35. IgG1 g:5411 36. IgG1 g:5510 37. IgG1 M.ox Norm Resp 38. IgG1 Ungly 39. IgG1 Ungly Norm Resp 40. IgG1 Norm Resp 41. IgG2 g:3410 42. IgG2 g:3510 43. IgG2 g:4400 44. IgG2 g:4410 45. IgG2 g:4411 | 1. IgG2 g:4500 2. IgG2 g:4510 3. IgG2 g:5411 4. IgG2 g:5510 5. IgG2 Norm Resp 6. IgG3 Norm Resp 7. IgG3/4 g:3510 8. IgG3/4 g:4410 9. IgG3/4 g:4411 10. IgG3/4 g:4510 11. IgG4 Norm Resp 12. IgM (mg/mL) 13. IgM p:205 g:5512 14. IgM p:209 g:4511 15. IgM p:209 g:5411 16. IgM p:209 g:5412 17. IgM p:209 g:5511 18. IgM p:209 g:5512 19. IgM p:439 g:5200 20. IgM p:439 g:6200 21. IgM p:439 g:7200 22. IgM p:439 g:8200 23. IgM p:439 g:9200 24. IgM p:46 g:4311 25. IgM p:46 g:5411 26. IgM p:46 g:5412 27. IgM p:46 g:5501 28. IgM p:46 g:5502 29. IgM p:46 g:5511 30. IgM p:46 g:5601 31. IgM J g:5401 32. IgM J g:5411 33. IgM J g:5412 34. Relative IgM 35. IgA1/2 36. IgM p:439 Ungly 37. IgG3/4 38. TF (mg/mL) 39. TF p:432 g:5402 40. TF p:432 g:5412 41. TF p:432 g:6502 42. TF p:630 g:5401 43. TF p:630 g:5402 44. TF p:630 g:5412 45. TF p:630 g:6513 |
| --- | --- | --- | --- |

^a^Ungly denotes the lack of a glycan at the conserved CH-2 84.4 glycosylation site of Ig (immunoglobulin).

^b^A.off indicates an ApoC3 variant lacking its terminal alanine.

| **Supplementary Table 3- Glycan Structures** | |
| --- | --- |
| **Composition** | **Structure** |
| **3500** | **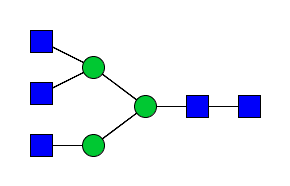** |
| **4401** | **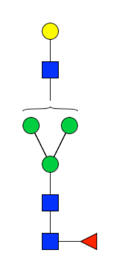** |
| **4500** | **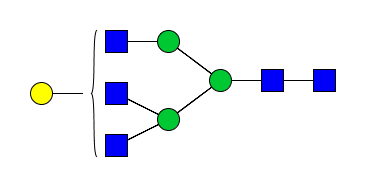** |
| **4501** | **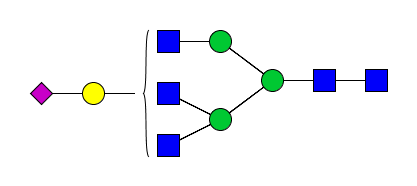** |
| **4510** | **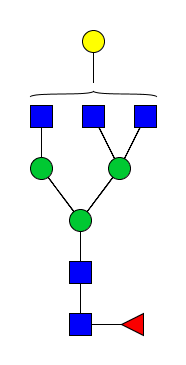** |
| **5200** | **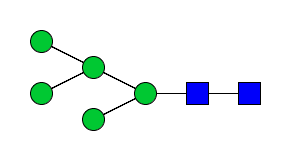** |
| **5400** | **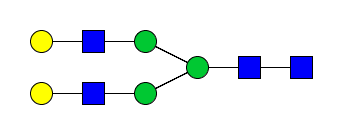** |
| **5401** | **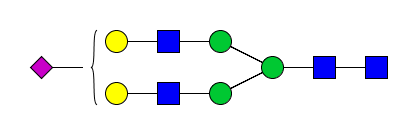** |
| **5402** | **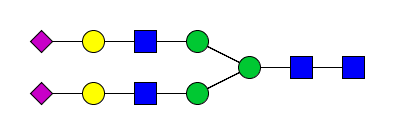** |
| **5410** | **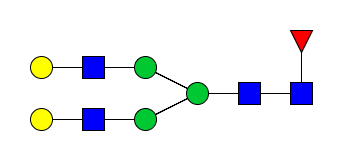** |
| **5411** | **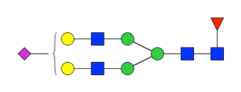** |
| **5412** | **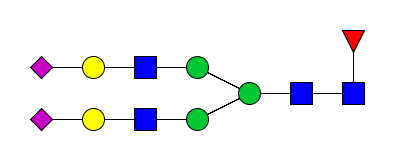** |
| **5500** | **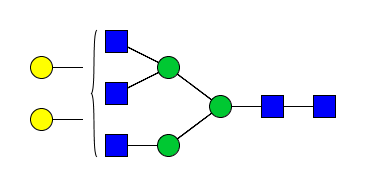** |
| **5501** | **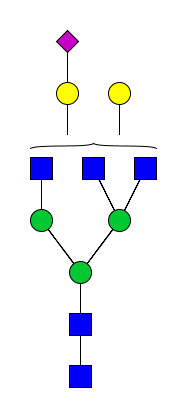** |
| **5502** | **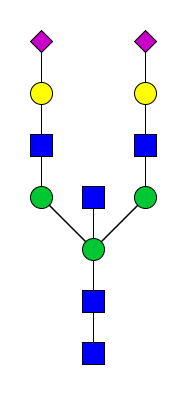** |
| **5510** | **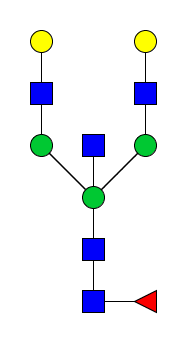** |
| **5511** | **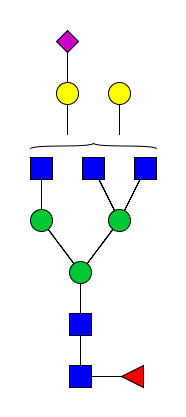** |
| **5512** | **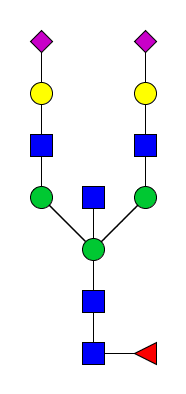** |
| **6200** | **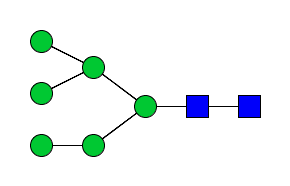** |
| **6501** | **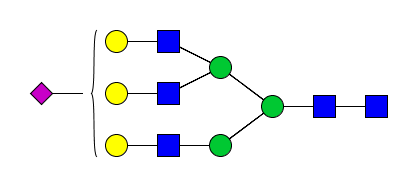** |
| **6502** | **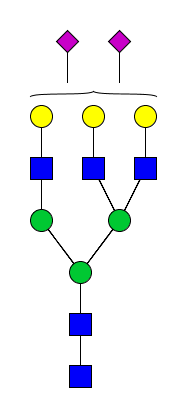** |
| **6503** | **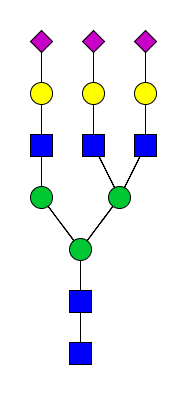** |
| **6512** | **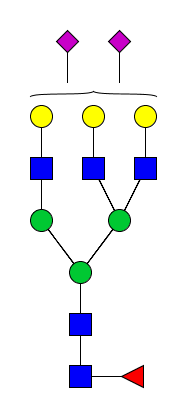** |
| **6513** | **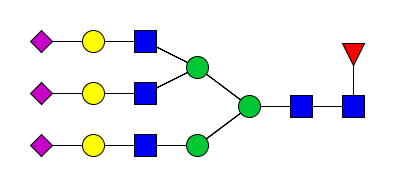** |
| **7200** | **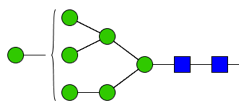** |
| **7602** | **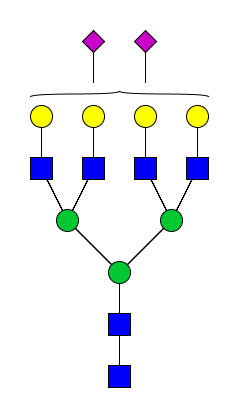** |
| **7603** | **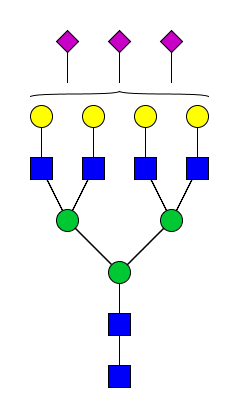** |
| **7604** | **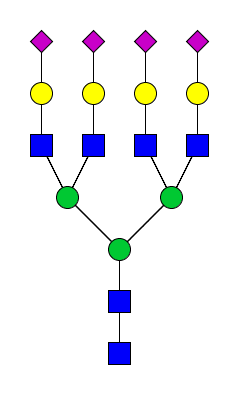** |
| **7613** | **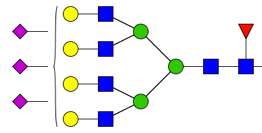** |
| **7614** | **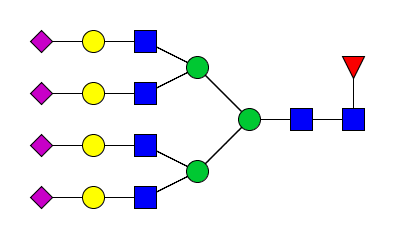** |
| **0300** | **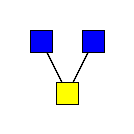** |
| **0310** | **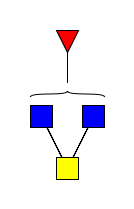** |
| **1101** | **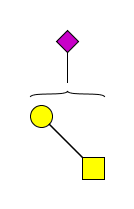** |
| **1102** | **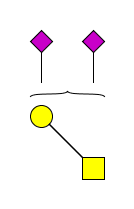** |
| **1111** | **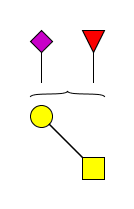** |
| **1201** | **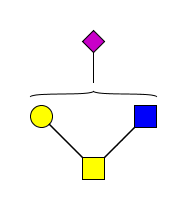** |
| **1202** | **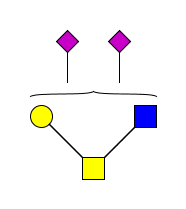** |
| **1210** | **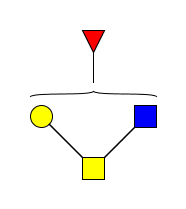** |
| **1300** | **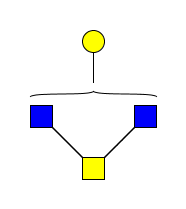** |
| **1311** | **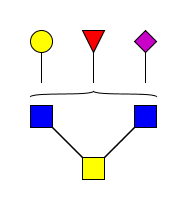** |
| **2200** | **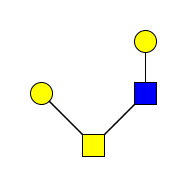** |
| **2211** | **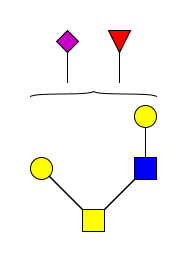** |
| **2212** | **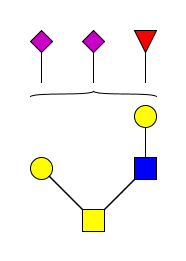** |
| **2220** | **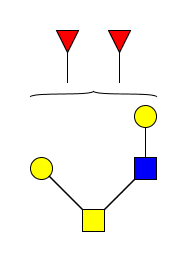** |
| **2221** | **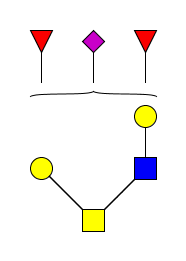** |
| **2230** | **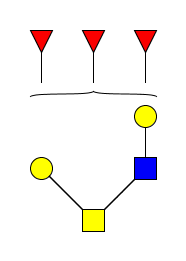** |

**Supplementary Table 4. Analytes** altered by age

| Analyte | ANCOVA  *P* value | ANCOVA  FDR^a^ |
| --- | --- | --- |
| A2HSG (mg/mL) | 0.00087 | 0.00782 |
| A2HSG p:156 g:5402 | 0.01152 | 0.04814 |
| A2HSG p:156 g:5412 | 6.1e-06 | 0.00016 |
| A2HSG p:156 g:5421 | 0.01190 | 0.04814 |
| A2HSG p:156 g:6503 | 0.00544 | 0.02913 |
| A2HSG p:156 g:6513 | 0.00746 | 0.03572 |
| A2HSG p:176 g:5402 | 0.00389 | 0.02284 |
| A2HSG p:176 g:5412 | 0.00659 | 0.03329 |
| A2HSG p:176 g:5431 | 0.00971 | 0.04450 |
| A2HSG p:176 g:7600 | 0.01186 | 0.04814 |
| A2HSG p:346 g:1101 | 0.00046 | 0.00493 |
| A2HSG p:346 g:2200 | 0.00074 | 0.00705 |
| ApoC3 p:74 g:1102 | 0.01004 | 0.04455 |
| HP p:207 g:121015 | 0.00015 | 0.00192 |
| IgA1/2 p:144 g:4401 | 0.00717 | 0.03529 |
| IgA1/2 p:144 g:4500 | 1.1e-06 | 3.3e-05 |
| IgA1/2 p:144 g:4501 | 0.00032 | 0.00390 |
| IgA1/2 p:144 g:5401 | 0.00631 | 0.03279 |
| IgA1/2 p:144 g:5402 | 5.4e-05 | 0.00089 |
| IgA1/2 p:144 g:5500 | 0.00090 | 0.00782 |
| IgA2 p:205 g:5412 | 0.00978 | 0.04450 |
| IgA2 p:205 g:5510 | 4.1e-05 | 0.00074 |
| IgA2 p:205 g:5511 | 0.00106 | 0.00837 |
| IgG1 Norm Resp | 0.00259 | 0.01630 |
| IgG1 g:3410 | 0.00036 | 0.00405 |
| IgG1 g:3510 | 1.4e-05 | 0.00032 |
| IgG1 g:5400 | 0.00201 | 0.01410 |
| IgG1 g:5410 | 3.2e-09 | 5.9e-07 |
| IgG1 g:5411 | 3.8e-07 | 1.6e-05 |
| IgG1 g:5510 | 0.00513 | 0.02828 |
| IgG2 g:3410 | 4.5e-07 | 1.6e-05 |
| IgG2 g:3510 | 3.8e-07 | 1.6e-05 |
| IgG2 g:5411 | 0.00013 | 0.00181 |
| IgM (mg/mL) | 0.00146 | 0.01105 |
| IgM p:209 g:4511 | 0.00186 | 0.01358 |
| IgM p:209 g:5411 | 7.9e-08 | 7.2e-06 |
| IgM p:209 g:5412 | 0.00404 | 0.02296 |
| IgM p:46 g:5412 | 0.00220 | 0.01484 |
| IgM p:46 g:5502 | 0.01053 | 0.04562 |
| IgM p:46 g:5601 | 3.1e-05 | 0.00062 |
| IgM J g:5401 | 0.00260 | 0.01630 |
| IgM J g:5412 | 0.00050 | 0.00503 |
| Relative IgM | 0.00286 | 0.01732 |
| IgM p:439 Ungly | 0.00010 | 0.00156 |
| TF p:630 g:6513 | 0.00098 | 0.00810 |

^a^False discovery rate.

*ANCOVA (analysis of covariance).

**Supplementary Table 5. Analytes altered by gender**

| Analyte | ANCOVA  *P* value | ANCOVA  FDR^a^ |
| --- | --- | --- |
| A1AT p:271 g:5412 | 0.00023 | 0.012 |
| A2HSG (mg/mL) | 0.00032 | 0.012 |
| A2HSG p:156 g:5401 | 0.00400 | 0.045 |
| A2HSG p:346 g:1101 | 0.00063 | 0.016 |
| A2HSG p:346 g:2200 | 0.00063 | 0.016 |
| A2MG (mg/mL) | 0.00012 | 0.012 |
| A2MG p:1424 g:5411 | 0.00293 | 0.039 |
| AGP1 p:103 g:7602 | 0.00027 | 0.012 |
| AGP12 p:56 g:6502 | 0.00212 | 0.039 |
| AGP12 p:56 g:6503 | 0.00084 | 0.019 |
| Hp p:184 g:6502 | 0.00105 | 0.021 |
| Hp p:207 g:10804 | 0.00298 | 0.039 |
| Hp p:207 g:11904 | 0.00435 | 0.047 |
| IgA12 p:144 g:5501 | 0.00239 | 0.039 |
| IgM (mg/mL) | 0.00014 | 0.012 |
| Relative IgM | 0.00342 | 0.041 |
| IgM p:439 Ungly | 0.00285 | 0.039 |
| ^a^False discovery rate.  *ANCOVA (analysis of covariance). | | |

| **Supplementary Table 6. Proteins altered by gender** | | | | |
| --- | --- | --- | --- | --- |
| Analyte conc.  (mg/mL) | Female | Male | *P* value | FDR^a^ |
| A1AT | 0.96 ± 0.3 | 0.81 ± 0.2 | 0.00522 | 0.053 |
| A2HSG | 0.44 ± 0.3 | 0.25 ± 0.1 | 0.00032 | 0.012 |
| A2MG | 1.3 ± 0.4 | 1 ± 0.3 | 0.00012 | 0.012 |
| IgM | 0.87 ± 0.6 | 0.49 ± 0.2 | 0.00014 | 0.012 |

^a^False discovery rate

**Supplementary Table 7. Multiple linear regression models for age prediction**

| Glycan only model | | | | | | |
| --- | --- | --- | --- | --- | --- | --- |
|  | COEFF^a^ | p value | VIF^b^ | ANCOVA^c^  p value | | ANCOVA  FDR^d^ |
| Intercept | 108.35 | <2e-16 |  |  | |  |
| IgG1 g:3510 | 9.37 | 8.0e-8 | 1.29 | 1.4e-5 | | 3.2e-4 |
| IgG1 g:5410 | -2.82 | 2.4e-5 | 1.51 | 3.2e-9 | | 5.9e-7 |
| IgM p:209 g:5411 | -257.57 | 1.9e-3 | 1.43 | 7.9e-8 | | 7.2e-6 |
| IgM J g:5411 | 23.48 | 1.0e-5 | 1.43 | 5.0e-4 | | 5.0e-3 |
| Hp p:241 g:7602 | 22.56 | 1.1e-5 | 1.04 | 1.6e-2 | | 0.063 |
| Glycans only model: 5-fold cross validation test performance | | | | | | |
| RMSE^e^ | R^2f^ | RMSE SD | | | R^2^ SD | |
| 8.65 | 0.62 | 1.16 | | | 0.12 | |
|  | | | | | | |

| Combined model | | | | | |  |
| --- | --- | --- | --- | --- | --- | --- |
|  | COEFF^a^ | p value | VIF^b^ | ANCOVA^c^  p value | ANCOVA  FDR^d^ |  |
| Intercept | 82.74 | 1.0e-12 |  |  |  |  |
| IgG3 Norm Resp | -13.46 | 3.8e-4 | 1.10 | 4.5e-2 | 0.14 |  |
| IgG1 g:3510 | 5.31 | 8.3e-3 | 2.35 | 1.4e-5 | 3.2e-4 |  |
| IgG1 g:5410 | -1.34 | 4.9e-2 | 2.06 | 3.2 e-9 | 5.9e-7 |  |
| IgG2 g:3410 | 1.69 | 8.3e-4 | 2.29 | 4.5e-7 | 1.6e-5 |  |
| IgM p:209 g:5411 | -335.93 | 2.4e-5 | 1.52 | 7.9e-8 | 7.2e-6 |  |
| IgM J g:5412 | 27.92 | 5.6e-8 | 1.52 | 5.0e-4 | 5.0e-3 |  |
| Hp p:241 g:7602 | 20.91 | 7.8e-6 | 1.05 | 1.6e-2 | 0.063 |  |
| Combined model: 5-fold cross validation test performance | | | | | |  |
| RMSE^e^ | R^2f^ | RMSE SD | R^2^ SD | | |  |
| 8.21 | 0.67 | 0.48 | 0.05 | | |  |
| ^a^Coefficient. ^b^Variance inflation factor (VIF). ^c^ANCOVA (analysis of covariance). ^d^FDR (false discovery rate),  ^e^Root-mean-square error. ^f^Coefficient of determination. | | | | | | |

| **Supplementary Table 8. Age prediction models with increasing number of predictors** | | | | | |
| --- | --- | --- | --- | --- | --- |
| Glycans only model | | | | | |
| Number of predictors | Predictors | RMSE^a^ | R^2b^ | RMSE  SD | R^2^  SD |
| 1 | IgG1 g:5410 | 11.76 | 0.32 | 1.32 | 0.14 |
| 2 | IgG1 g:3510 + IgG1 g:5410 | 10.05 | 0.51 | 1.34 | 0.11 |
| 3 | IgG1 g:3510 + IgG1 g:5410 + HP p:241 g:7602 | 9.43 | 0.54 | 1.30 | 0.16 |
| 4 | IgG1 g:3510 + IgG1 g:5410 + IgM J g:5412 + HP p:241 g:7602 | 8.70 | 0.60 | 1.76 | 0.17 |
| 5 | IgG1 g:3510 + IgG1 g:5410 + IgM p:209 g:5411 + IgM J g:5412 + HP p:241 g:7602 | 8.65 | 0.62 | 1.16 | 0.12 |
| Combined model | | | | | |
| Number of predictors | Predictors | RMSE^a^ | R^2b^ | RMSE  SD | R^2^  SD |
| 1 | IgG1 g:5410 | 11.76 | 0.32 | 1.32 | 0.14 |
| 2 | IgG1 g:3510 + IgG1 g:5410 | 10.05 | 0.51 | 1.34 | 0.11 |
| 3 | IgG1 g:3510 + IgG1 g:5410 + Hp p:241 g:7602 | 9.43 | 0.54 | 1.30 | 0.16 |
| 4 | IgG1 g:3510 + IgG1 g:5410 + IgM J g:5412 + Hp p:241 g:7602 | 8.70 | 0.60 | 1.76 | 0.17 |
| 5 | IgG1 g:3510 + IgG1 g:5410 + IgM p:209 g:5411 + IgM J g:5412 + Hp p:241 g:7602 | 8.65 | 0.62 | 1.16 | 0.12 |
| 6 | IgG3 Norm Resp + IgG1 g:3510 + IgG1 g:5410 + IgM p:209 g:5411 + IgM J g:5412 + Hp p:241 g:7602 | 8.44 | 0.66 | 0.76 | 0.09 |
| 7 | IgG3 Norm Resp + IgG1 g:3510 + IgG1 g:5410 + IgG2 g:3410 + IgM p:209 g:5411 +  IgM J g: 5412 + Hp p:241 g:7602 | 8.21 | 0.67 | 0.48 | 0.05 |

^a^Root-mean-square error. ^b^Coefficient of determination.
